# Extracellular vesicles derived from Wharton’s Jelly mesenchymal stem cells inhibit the tumor environment via the miR-125b/HIF1α signaling pathway

**DOI:** 10.1038/s41598-022-17767-y

**Published:** 2022-08-08

**Authors:** Yun-Hsuan Chang, Cat-Khanh Vuong, Nhat-Hoang Ngo, Toshiharu Yamashita, Xiucai Ye, Yasunori Futamura, Mizuho Fukushige, Mana Obata-Yasuoka, Hiromi Hamada, Motoo Osaka, Yuji Hiramatsu, Tetsuya Sakurai, Osamu Ohneda

**Affiliations:** 1 Ph.D. Program in Humanics, University of Tsukuba, 1-1-1 Tennodai, Tsukuba, Ibaraki 305-8575 Japan; 2 Graduate School of Comprehensive Human Science, Laboratory of Regenerative Medicine and Stem Cell Biology, University of Tsukuba, 1-1-1 Tennodai, Tsukuba, Ibaraki 305-8575 Japan; 3 Department of Computer Science, University of Tsukuba, 1-1-1 Tennodai, Tsukuba, Ibaraki 305-8575 Japan; 4 Department of Obstetrics and Gynecology, University of Tsukuba, Tsukuba, Japan; 5 Department of Cardiovascular Surgery, University of Tsukuba, Tsukuba, Japan

**Keywords:** Breast cancer, Mesenchymal stem cells, Computational biology and bioinformatics

## Abstract

Triple negative breast cancer (TNBC) is associated with worse outcomes and results in high mortality; therefore, great efforts are required to find effective treatment. In the present study, we suggested a novel strategy to treat TNBC using mesenchymal stem cell (MSC)-derived extracellular vesicles (EV) to transform the behaviors and cellular communication of TNBC cells (BCC) with other non-cancer cells related to tumorigenesis and metastasis. Our data showed that, BCC after being internalized with EV derived from Wharton’s Jelly MSC (WJ-EV) showed the impaired proliferation, stemness properties, tumorigenesis and metastasis under hypoxic conditions. Moreover, these inhibitory effects may be involved in the transfer of miRNA-125b from WJ-EV to BCC, which downregulated the expression of HIF1α and target genes related to proliferation, epithelial-mesenchymal transition, and angiogenesis. Of note, WJ-EV-internalized BCC (wBCC) showed transformed behaviors that attenuated the in vivo development and metastatic ability of TNBC, the angiogenic abilities of endothelial cells and endothelial progenitor cells and the generation of cancer-associated fibroblasts from MSC. Furthermore, wBCC generated a new EV with modified functions that contributed to the inhibitory effects on tumorigenesis and metastasis of TNBC. Taken together, our findings suggested that WJ-EV treatment is a promising therapy that results in the generation of wBCC to interrupt the cellular crosstalk in the tumor environment and inhibit the tumor progression in TNBC.

## Introduction

Triple negative breast cancer (TNBC) is an aggressive type of breast cancer which is highly metastatic and challenging to treat^1^. Chemotherapy remains the standard of care for TNBC; however, it is associated with limitations, including a short treatment response followed by rapid relapse and metastasis^2,3^. Thus, there is an urgent need to identify an effective treatment for TNBC.

Inside the tumor, cancer cells interact and transform numerous types of non-cancer cells to create a facilitated environment for tumor development and metastasis^4^. For example, cancer cells activate endothelial cells (EC) and endothelial progenitor cells (EPC) to start angiogenesis and neovascularization to supply the nutrients and provide a route for the invasion of cancer cells^5–7^. Furthermore, cancer cells induce the generation of cancer associated fibroblasts (CAF) from their progenitors, such as mesenchymal stem cells, which promote the proliferation, epithelial-mesenchymal transition (EMT), and metastasis of cancer cells^8–11^. Therefore, several studies have suggested that modifying the cellular behaviors inside the tumor may be a novel strategy to find an effective treatment for aggressive cancers, such as TNBC^12–14^.

Recent studies have suggested the application of extracellular vesicles (EV) derived from mesenchymal stem cells (MSC) as a promising agent for cell-free therapy for numerous diseases^15–17^. EV are a population of cell-derived membrane vesicles that carry biological messages, including proteins, mRNAs, and miRNAs, which can internalize to the target cells and modulate the genotype and phenotype of these recipient cells^18–20^. EV therefore serve as a promising candidate to modify cellular behaviors in cancer therapy. In particular, several studies have suggested that MSC-EV are involved in inhibiting tumorigenesis and downregulating the expression of angiogenic genes in recipient cells^15,16,20^. However, the effects of MSC-EV on breast cancer cells remains controversial. We previously showed that adipose tissue MSC-EV promoted metastasis in TNBC cells (BCC)^21^, while the other study reported that Wharton Jelly’s MSC-derived secretome, which contains EV, inhibits the tumor progression of breast cancer^22^. These findings suggest that the effects of MSC-EV on breast cancer might be related to the source of tissues from which MSC are isolated. Therefore, finding an appropriate source of MSC-EV that inhibits BCC is important for developing an effective treatment for TNBC.

In the present study, we aimed to modify the cellular behaviors of BCC to inhibit breast tumor progression and metastasis by the internalization of BCC with MSC-EV. The tumorigenic characteristics and the effects of BCC internalized with MSC-EV on several types of cells which are involved in the tumor progression and metastasis in the tumor environment, were determined. Our findings suggested that after being internalized with MSC-EV from Wharton’s Jelly (WJ-EV), BCC showed the transformed phenotypes which attenuated the development and metastasis of TNBC.

## Results

### EV from Wharton’s Jelly MSC impaired the tumorigenic and metastatic abilities of breast cancer cells (BCC)

**Figure 1:**
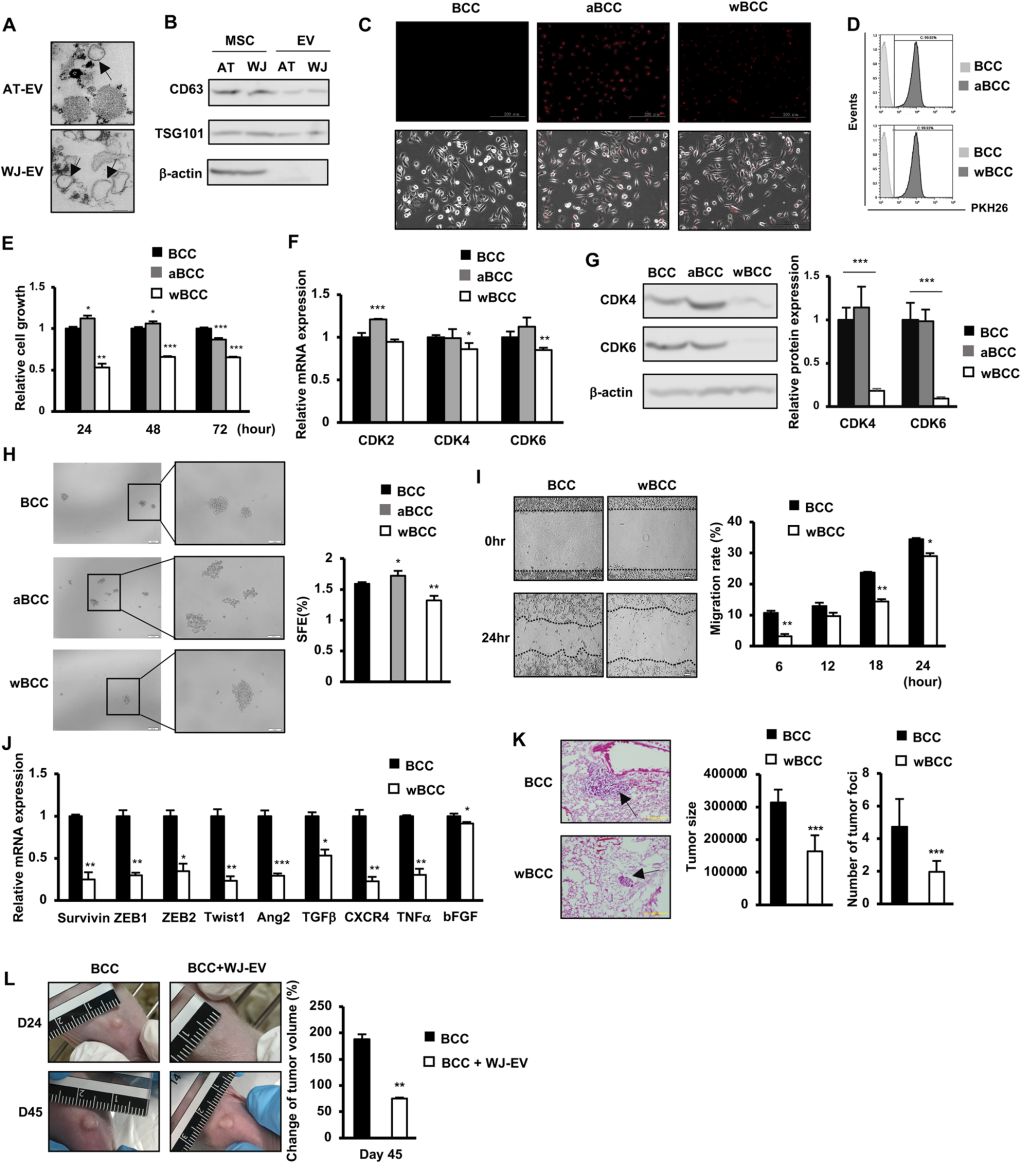
WJ-EV impaired the tumorigenic and metastatic abilities of BCC. (**A**) The morphology of AT-EV and WJ-EV. (**B**) Markers of AT-EV and WJ-EV, n = 3. Full-length blots were shown in Supplementary Fig. S3A. (**C**) The internalization of PKH26-stained AT-EV and WJ-EV into BCC; aBCC, AT-EV-internalized BCC; wBCC, WJ-EV-internalized BCC, n = 3. Images were taken at × 20 magnification. (**D**) The internalization of AT-EV and WJ-EV into BCC were examined by a flow cytometry, n = 3. (**E**) The cellular proliferation of AT-EV- and WJ-EV-internalized BCC; aBCC, AT-EV-internalized BCC; wBCC, WJ-EV-internalized BCC, n = 3, *p < 0.05, **p < 0.01, ***p < 0.001. (**F**) The expression of genes involving in proliferation of BCC, aBCC and wBCC, n = 3, *p < 0.05, **p < 0.01, ***p < 0.001. (**G**) The protein expression of CDK4 and CDK6 in BCC, aBCC and wBCC, n = 3, ***p < 0.001. Full-length blots were shown in Supplementary Fig. S3B. (**H**) The sphere formation of BCC and wBCC, n = 3, *p < 0.05, **p < 0.01. Images were taken at × 4 and × 10 magnification. (**I**) The migration assay of BCC and wBCC, n = 3, *p < 0.05, **p < 0.01. (**J**) The expression of genes related to EMT and migration in BCC and wBCC, n = 3, *p < 0.05, **p < 0.01, ***p < 0.001. (**K**) The metastatic ability of BCC and wBCC in vivo. n = 4, ***p < 0.001. (**L**) The effects of WJ-EV treatment on tumor growth of BCC in vivo. n = 4, **p < 0.01. Raw images of tumors were shown in Supplementary Fig. S4A. All experiments were performed in triplicate.

First, in order to examine the effects of MSC-EV on the tumorigenesis of BCC, EV were isolated from adipose tissue-derived MSC (AT-EV) and Wharton’s Jelly-derived MSC (WJ-EV). The isolated AT-EV and WJ-EV both showed a round shape (diameter, 200 nm) (Fig. 1A) and expressed biomarkers for EV (positive for CD63 and TSG101; negative for β-actin) (Fig. 1B). In addition, observation of the internalization of EV to BCC showed that after 24 h of treatment, almost all BCC in the population were incorporated with either AT-EV or WJ-EV (Fig. 1C,D).

As hypoxia plays a vital role in tumor progression, next, the proliferation, sphere formation and hypoxic responses under hypoxic conditions were characterized in AT-EV- or WJ-EV-internalized BCC. The results showed that while AT-EV slightly induced the proliferation of BCC, WJ-EV significantly impaired the proliferation of BCC (Fig. 1E), which might be involved in the downregulation of CDK4 and CDK6 at both mRNA and protein levels (Fig. 1F,G). In addition, the mammosphere assay showed that AT-EV promoted sphere formation, while WJ-EV reduced this function in BCC (Fig. 1H).

Metastasis is reported to be the primary cause of mortality in patients with breast cancer^3^; thus, we next examined the effects of WJ-EV on the metastatic abilities of BCC under hypoxic conditions. The results showed that in comparison to the original BCC, WJ-EV-internalized BCC (wBCC) showed a significantly reduced migration ability (1.7-fold decrease, Fig. 1I). Consistently, in comparison to BCC, wBCC showed the downregulation of genes related to the epithelial-mesenchymal transition (EMT) and the migration of cancer cells (e.g., *zeb1, zeb2, twist1,* and *tgfβ*)^23,24^ (Fig. 1J). Furthermore, the downregulated effects of WJ-EV on the expression of these genes were not temporary; rather, they remained in wBCC after sub-culture in which wBCC—after being passaged four times—still showed the impaired expression of these genes in comparison to BCC (Supplementary Fig. S1).

Next, we examined in vivo metastasis of wBCC in a lung metastasis mouse model via tail vein injection. The results showed that—consistent with the in vitro impaired migratory abilities—wBCC showed less ability to metastasize to the mouse lung, which was demonstrated by a lower number of tumor foci and smaller tumor size, in comparison to BCC (Fig. 1K). Furthermore, we examined the in vivo inhibitory effects of WJ-EV on breast cancer tumor growth in a tumor xenograft mouse model. We generated tumors by injecting BCC into the mammary fat pads of nude mice, then injected WJ-EV into the established tumors. The tumor volumes of mice injected with WJ-EV were compared to those without injection. As a result, the injection of WJ-EV into tumor sites significantly reduced the tumor volume in mice (2.5-fold decrease, Fig. 1L).

Taken together, these data suggested that WJ-EV, but not AT-EV, significantly impaired the proliferation, sphere formation, migration and EMT-related gene expression of BCC under hypoxic conditions, which were related to attenuated in vivo tumorigenesis and metastasis of BCC. wBCC showed transformed phenotypes that were different from the original BCC which might attenuate the development and metastasis of TNBC.

### wBCC showed a transformed function which impaired the tumorigenic abilities of cells in the tumor microenvironment

**Figure 2:**
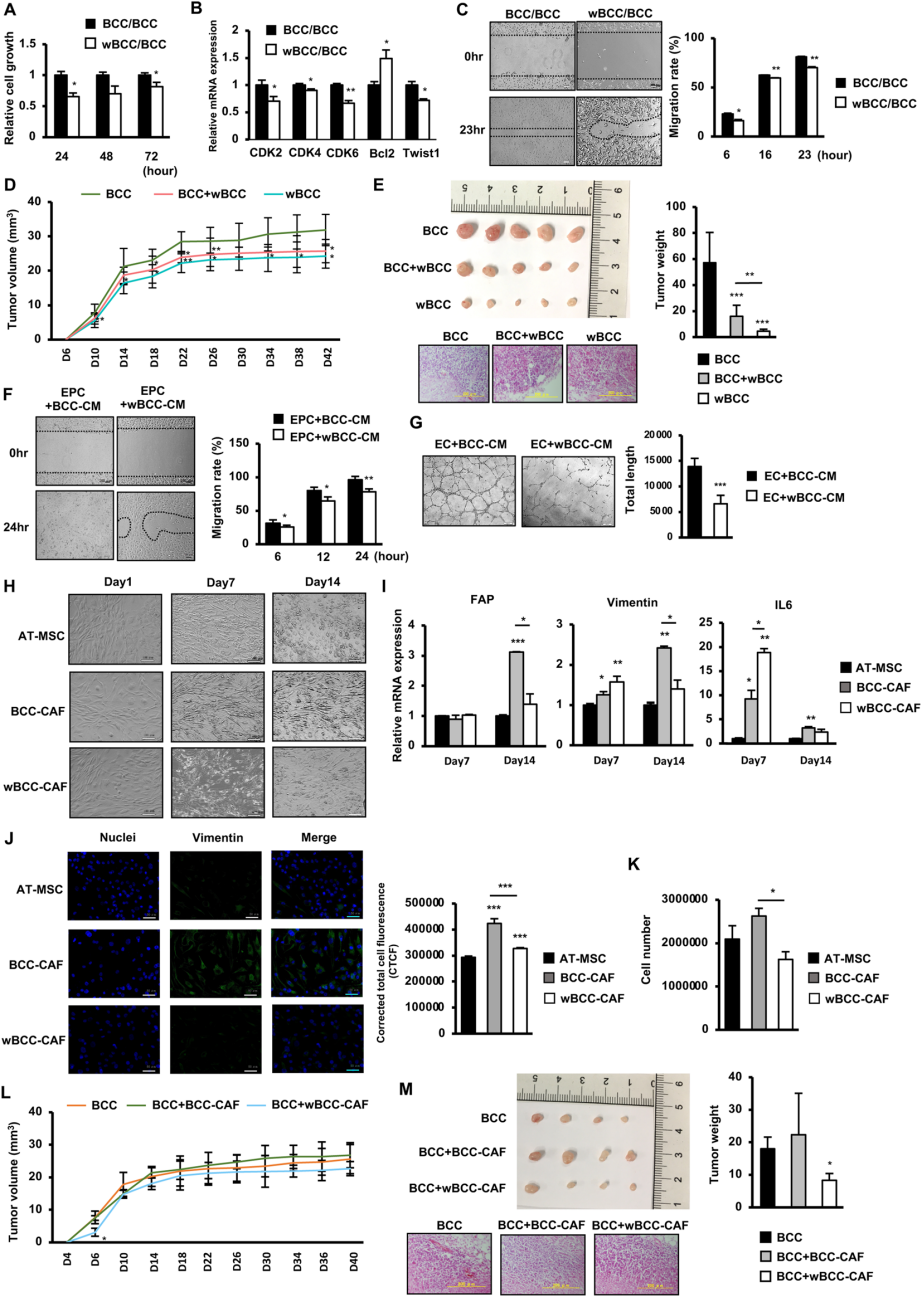
The impact of wBCC on the other cells in the tumor microenvironment. (**A**) The cell proliferation and (**B**) the gene expression of BCC cocultured with BCC or wBCC at ratio of 1:1 (cell count), n = 3, *p < 0.05, **p < 0.01. (**C**) The cell migration of BCC cocultured with BCC or wBCC at ratio of 1:1 (cell count), n = 3, *p < 0.05, **p < 0.01. (**D**) The tumor growth of BCC, BCC cocultured with wBCC (at ratio of 1:1), and wBCC in vivo*,* n = 8, *p < 0.05, **p < 0.01. (**E**) The tumor size and tumor weight of BCC, BCC cocultured with wBCC at ratio of 1:1 (cell count), and wBCC in vivo*,* n = 8, **p < 0.01, ***p < 0.001. Raw images of tumors were shown in Supplementary Fig. S4B. (**F**) The migration of EPC treated with condition medium (CM) derived from BCC and wBCC for 24 h, n = 3, *p < 0.05, **p < 0.01. (**G**) The tube formation of EC treated with condition medium (CM) derived from BCC and wBCC for 24 h, n = 3, ***p < 0.001. (**H**) The cell morphology of AT-MSC, AT-MSC cocultured with BCC (BCC-CAF) or wBCC (wBCC-CAF), n = 3. (**I**) The expression of fibroblast markers in AT-MSC, BCC-CAF or wBCC-CAF, n = 3, *p < 0.05, **p < 0.01, ***p < 0.001. (**J**) Immunofluorescence staining of AT-MSC, BCC-CAF or wBCC-CAF on Day 14 (green: Vimentin; blue: Nuclei), n = 3, ***p < 0.001. (**K**) The cell number of AT-MSC, BCC-CAF or wBCC-CAF on Day 14, n = 3, *p < 0.05. (**L**) The tumor growth in mice injected with BCC, BCC with BCC-CAF, or BCC with wBCC-CAF, n = 8, *p < 0.05. (**M**) The tumor size and tumor weight in mice injected with BCC, BCC with BCC-CAF, or BCC with wBCC-CAF, n = 8, *p < 0.05. Raw images of tumors were shown in Supplementary Fig. S4C. All experiments were performed in triplicate.

In order to determine the functions of wBCC in the tumor progression, we next examined the effects of wBCC on several types of cells located inside the tumor, including BCC, EC, EPC, and MSC under hypoxic conditions. Firstly, the effects of wBCC on the original BCC was examined by coculturing wBCC and BCC using a Transwell. The results show that the proliferation of BCC cocultured with wBCC was significantly impaired; this might be involved in the downregulation of genes related to cell cycle and the upregulation of apoptotic genes (Fig. 2A,B)^25^. In addition to the impaired proliferation, coculturing with wBCC attenuated the migratory ability of BCC (Fig. 2C). Furthermore, in comparison to BCC, wBCC exhibited a significantly lower tumorigenic ability, which was demonstrated by a decreased tumor volume (Fig. 2D) and tumor weight in injected mice (Fig. 2E). Interestingly, mice injected with the mixture of BCC and wBCC showed impaired tumorigenesis with a smaller tumor volume (Fig. 2D) and tumor weight (Fig. 2E), in comparison to those injected with BCC, suggesting that wBCC have inhibitory effects on the tumorigenic activities of the original BCC.

Next, we examined the effects of wBCC on EPC and EC, which are the key players in angiogenesis, an important process in tumor progression^6,26^. The migratory ability and tube formation ability (processes involved in angiogenesis) were examined in EPC and EC, in the presence of condition medium derived from BCC (BCC-CM) or wBCC (wBCC-CM). The results of a scratch assay showed that EPC cultured in the presence of wBCC-CM exhibited impaired migratory ability in comparison to those cultured in the presence of BCC-CM (Fig. 2F). In addition to EPC, EC cultured in the presence of wBCC-CM showed reduced angiogenic ability in a tube formation assay in comparison to those cultured in the presence of BCC-CM, (2.1-fold decrease, Fig. 2G). These data suggested that in comparison to BCC, wBCC were associated with impaired paracrine effects on EC and EPC, which might be highly related to tumor angiogenesis.

Previous studies reported that MSC play a vital role in supporting cancer cells, which is partially conducted by differentiation to cancer-associated fibroblasts (CAF), the largest heterogeneous cell population in the tumor microenvironment^27^. As breast tumors are mainly surrounded by adipose tissues, we next examined the effects of wBCC on the differentiation ability of adipose tissue-derived MSC (AT-MSC) to CAF by co-culturing of AT-MSC with wBCC for 14 days and characterized the fibroblastic properties. The results showed that in comparison to the control AT-MSC, AT-MSC cocultured with BCC (BCC-CAF) or wBCC (wBCC-CAF) both showed a transformed morphology, with spindle shapes (similar to fibroblast-like cells) after 14 days of coculture (Fig. 2H). In addition, in comparison to AT-MSC, BCC-CAF showed the high upregulation of genes related to CAF differentiation (e.g., IL6 [3.23-fold increase]; Fig. 2I), and fibroblastic cell markers (e.g., FAP [3.11-fold increase] and vimentin [2.41-fold increase]; Fig. 2I). Interestingly, wBCC-CAF showed the less upregulation of these genes in comparison to BCC-CAF (Fig. 2I). Consistently, the immunostaining results showed that wBCC-CAF exhibited the lower expression of vimentin protein, a fibroblast marker, in comparison to BCC-CAF (Fig. 2J). In addition, wBCC-CAF showed impaired proliferation with a lower cell count in comparison to BCC-CAF (Fig. 2K). These data suggested that the impaired ability of wBCC to induce the differentiation of AT-MSC to CAF rather than BCC.

We next compared the functions of BCC-CAF and wBCC-CAF to support the in vivo tumorigenesis of BCC in the tumor xenograft mouse model. BCC were cocultured with either BCC-CAF (BCC-CAF/BCC) or wBCC-CAF (wBCC-CAF/BCC) for 24 h in a Transwell before collection for injection into the mammary fat pads of nude mice. As a result, mice injected with BCC-CAF/BCC showed higher tumorigenesis, which generated larger tumors in comparison to those injected with BCC (Fig. 2L,M), suggesting that BCC-CAF induced the tumorigenic ability of BCC. Of note, mice injected with wBCC-CAF/BCC showed lower tumorigenesis with smaller generated tumors in comparison to those injected with BCC-CAF/BCC or BCC (Fig. 2L,M), suggesting that the ability to support the in vivo tumorigenesis of BCC may be impaired in wBCC-CAF.

Taken together, these data suggested that wBCC showed a transformed function, which may attenuate tumor progression. This transformed function is highly involved in the impaired paracrine effects, which promote the lower tumorigenic ability of cells inside the tumor microenvironment in comparison to the original BCC.

### wBCC generated transformed EV, which impaired the metastasis of BCC

**Figure 3:**
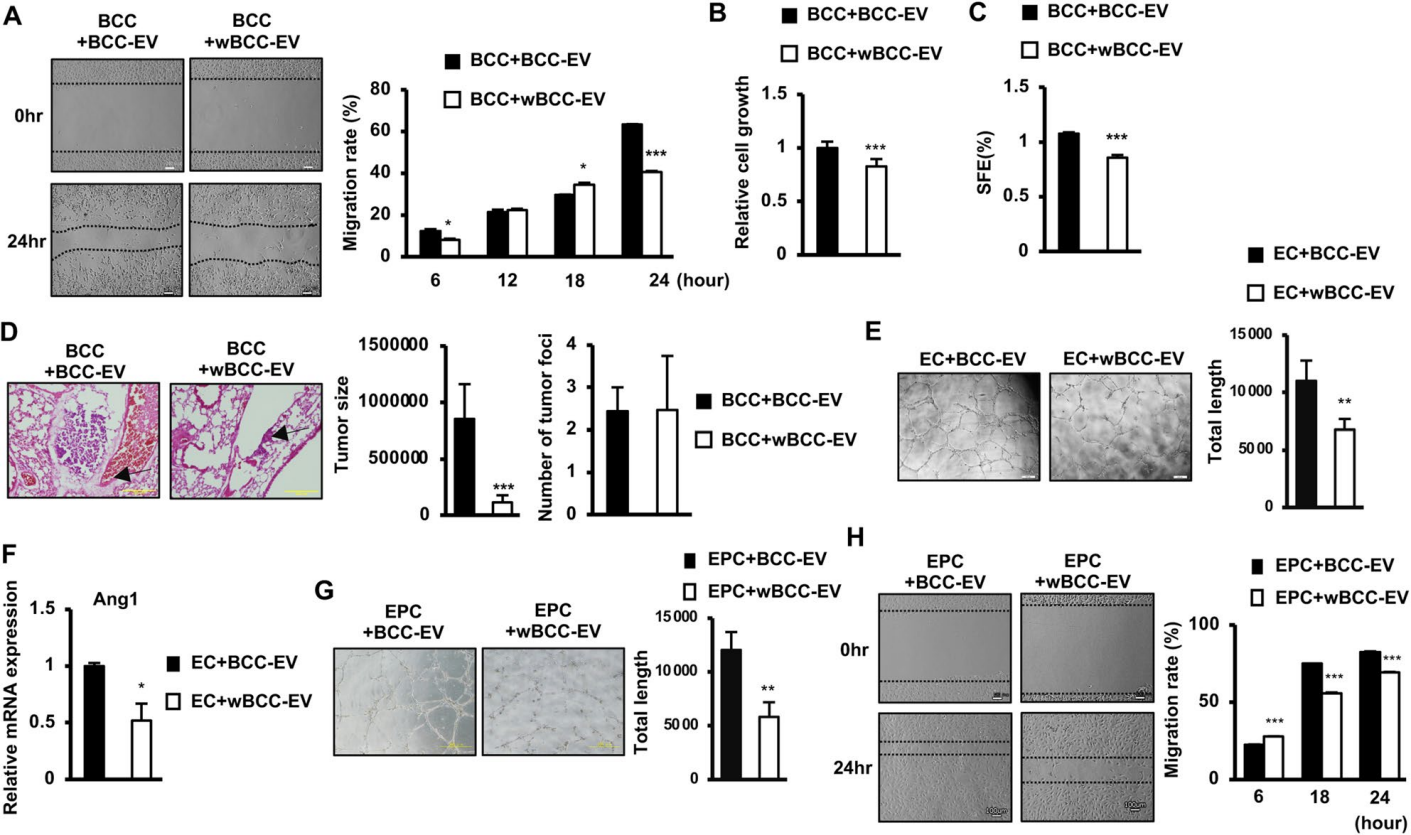
The effects of wBCC-EV on BCC, EC, and EPC. (**A**) The cell migration of BCC treated with BCC-EV or wBCC-EV, n = 3, *p < 0.05, ***p < 0.001. (**B**) The cell proliferation of BCC treated with BCC-EV or wBCC-EV for 24 h, n = 3, ***p < 0.001. (**C**) The sphere formation of BCC treated with BCC-EV or wBCC-EV for 24 h, n = 3, ***p < 0.001. (**D**) The tumor metastatic ability of the BCC treated with BCC-EV or wBCC-EV in vivo, n = 4, *p < 0.05, **p < 0.01, ***p < 0.001. (**E**) The tube formation of EC treated with BCC-EV or wBCC-EV, n = 9, **p < 0.01. (**F**) The angiogenic gene expression of EC treated with BCC-EV or wBCC-EV, n = 3, *p < 0.05. (**G**) The tube formation of EPC treated with BCC-EV or wBCC-EV, n = 9, **p < 0.01. (**H**) The cell migration of EPC treated with BCC-EV and wBCC-EV, n = 3, ***p < 0.001.

Due to the transformed characteristics and functions of wBCC, we next examined the functions of EV derived from wBCC (wBCC-EV) in comparison to those derived from the original BCC (BCC-EV). First, wBCC-EV and BCC-EV were isolated and internalized to BCC, then the characteristics of these internalized BCC were examined under hypoxic conditions. The results of a scratch assay showed that in comparison to BCC-EV-internalized BCC (BCC + BCC-EV), wBCC-EV-internalized BCC (BCC + wBCC-EV) showed an impaired migratory ability (Fig. 3A). In addition, the internalization of wBCC-EV reduced the growth of BCC, with BCC + wBCC-EV showing lower proliferation in comparison to BCC + BCC-EV (Fig. 3B). Moreover, the internalization of wBCC-EV impaired the stemness properties of BCC, in which BCC + wBCC-EV showed a lower number of generated spheres in a mammosphere assay in comparison to BCC + BCC-EV (Fig. 3C). Furthermore, the effect of wBCC-EV on the in vivo metastatic ability of BCC was examined. wBCC-EV or BCC-EV internalized BCC was intravenously injected to mice via the tail vein. The number of metastatic cancer cells in the mouse lung was examined. The results showed that the lungs of mice injected with BCC + wBCC-EV showed a similar number of tumor foci to those injected with BCC + BCC-EV, whereas the size of tumors in mice injected with BCC + wBCC-EV was significantly reduced (7.5-fold decrease, Fig. 3D).

Next, EC or EPC were internalized with wBCC-EV or BCC-EV, then the altered characteristics were examined under hypoxic conditions. The results showed that in comparison to BCC-EV, wBCC-EV reduced the tube formation ability of EC (Fig. 3E) which might be involved in the downregulation of the Ang1 expression (twofold downregulation, Fig. 3F), a factor that supports EC growth and induces sprout formation^28^. Moreover, besides EC, the functions of EPC which are involved in angiogenesis were also impaired by wBCC-EV, in comparison to BCC-EV-internalized EPC, wBCC-EV-internalized EPC showed reduced tube formation ability (Fig. 3G) and migration (Fig. 3H). Therefore, these data suggested that—in comparison to BCC-EV—the angiogenesis ability of wBCC-EV might be impaired in the tumor microenvironment.

Taken together, these results suggested that wBCC produced transformed EV, and that the ability of these transformed EV to regulate target cells—including cancer cells, EC, and EPC—was impaired, which might contribute to the attenuation of breast tumor progression and metastasis in the tumor microenvironment.

### wBCC showed an altered miRNA expression profile in comparison to BCC

**Figure 4:**
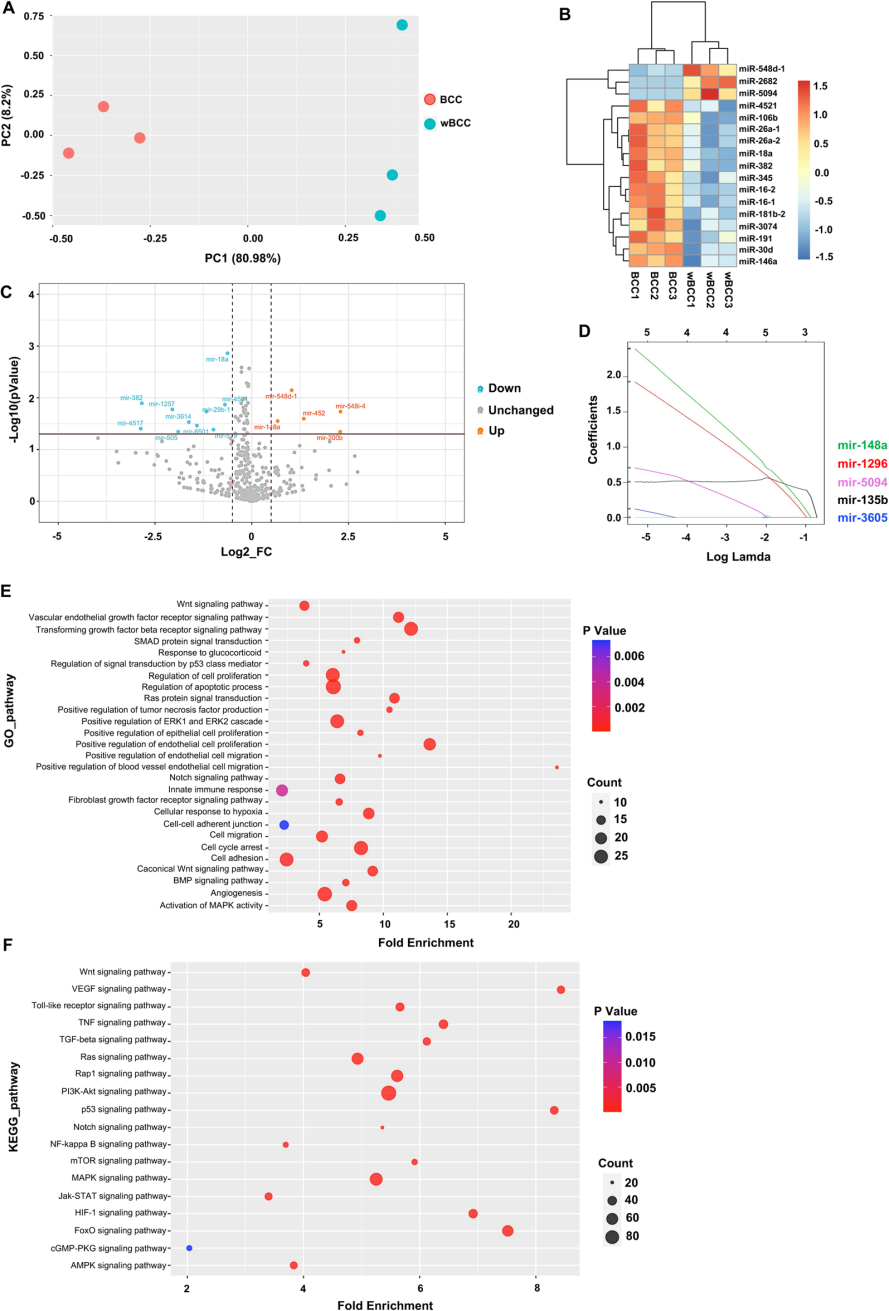
miRNA profile in the BCC and wBCC, as determined by a microRNA sequencing analysis. (**A**) The principal component analysis (PCA) of BCC and wBCC. (**B**) The expression of miRNAs in BCC and wBCC presented by a heatmap. (**C**) The differential expression of miRNAs in BCC and wBCC presented by volcano plot. (**D**) The key miRNAs were analyzed by least absolute shrinkage and selection operator (LASSO) regression. (**E**) The Gene Ontology (GO) pathway analysis to determine mRNAs targeted by miRNAs. (**F**) The Kyoto Encyclopedia of Genes and Genomes (KEGG) pathway analysis to determine mRNAs targeted by miRNAs^35–37^.

As recent studies reported the crucial roles of microRNA (miRNA) in the regulation of cell behavior^19,20^, we next performed miRNA sequencing to compare the miRNA profile of wBCC and BCC. First, the characteristics of BCC and wBCC were investigated by principal component analysis (PCA). The results showed that although the cluster of BCC and wBCC are not well separated according to PC2 (8.2%), which might be due to the limited number of samples, the cluster of BCC and wBCC showed separation according to PC1 (80.98%), suggesting that the characteristics of BCCs and wBCC can be separated (Fig. 4A). To investigate the different expression of miRNAs in BCC and wBCC, 15 miRNAs with significantly differentially expressed (*p* < 0.01) were examined by hierarchical clustering (Fig. 4B). As a result, the expression levels of mir-548d-1, mir-2682, and mir-5094 were found to be relatively higher in wBCC. Among these three miRNAs, mir-2682 and mir-5094 were previously suggested to be a tumor suppressor and a proliferation suppressor, respectively^29,30^.

Next, a total of 417 miRNAs for which the fold change of normalized expression value > 0.5, with a p value of < 0.05 between the BCC and wBCC groups were classified as upregulated or regulated in wBCC (Fig. 4C). The results showed that 5 miRNAs (mir-548i-4, mir-200b, mir-452, mir-548d-1, and mir-148a) were upregulated in wBCC, while 10 miRNAs (mir-18a, mir-4521, mir-579, mir-29b-1, mir-6501, mir-3614, mir-505, mir-382, mir-1257 and mir-4517) were downregulated in wBCC (Fig. 4C). Among the upregulated miRNAs in wBCC, miR-548 family and miR-452 were suggested to inhibit tumor development and metastasis in breast and lung cancer^19,31^. In addition, two studies reported that miR-200b and mir-148a inhibited tumor growth and metastasis in the TNBC cell line^32,33^.

To investigate the key miRNAs in the inhibition of tumor development, we further analyzed the expression of miRNAs in both BCC and wBCC by least absolute shrinkage and selection operator (LASSO) regression. After normalization, a total number of 609 features (miRNAs) were computed and feature selection were conducted by the LASSO regression algorithm. Based on the results, 5 miRNAs (mir-148a, mir-1296, mir-5094, mir-135b, and mir-3605) were regarded as promising key factors in the inhibition of wBCC (Fig. 4D). Previous studies reported the roles of mir-148a, mir-1296, and mir-5094 as tumor suppressors^30,33,34^. In particular, mir-148a and mir-1296 were regarded as tumor suppressors in the TNBC cell line^33,34^. In addition, the signaling pathways associated with the development of BCC and wBCC were determined by an ingenuity pathway analysis (IPA) using a gene ontology analysis and a KEGG analysis^35–37^. These analyses detected miRNAs targeting mRNAs involved in several pathways related to tumor development and tumor metastasis (Fig. 4E,F). Notably, the KEGG pathway analysis showed that wBCC contain miRNAs targeting mRNAs were involved in the HIF1α signaling pathway (Fig. 4F).

Taken together, these data suggested that in comparison to BCC, wBCC showed an altered miRNA profile, which might be involved in the alteration of characteristics that are associated with tumorigenesis and metastasis of BCC under hypoxic conditions.

### miR-125b-derived from WJ-EV downregulated the expression of HIF1α in wBCC under hypoxic conditions, which contributed to transformation of the wBCC phenotype

**Figure 5:**
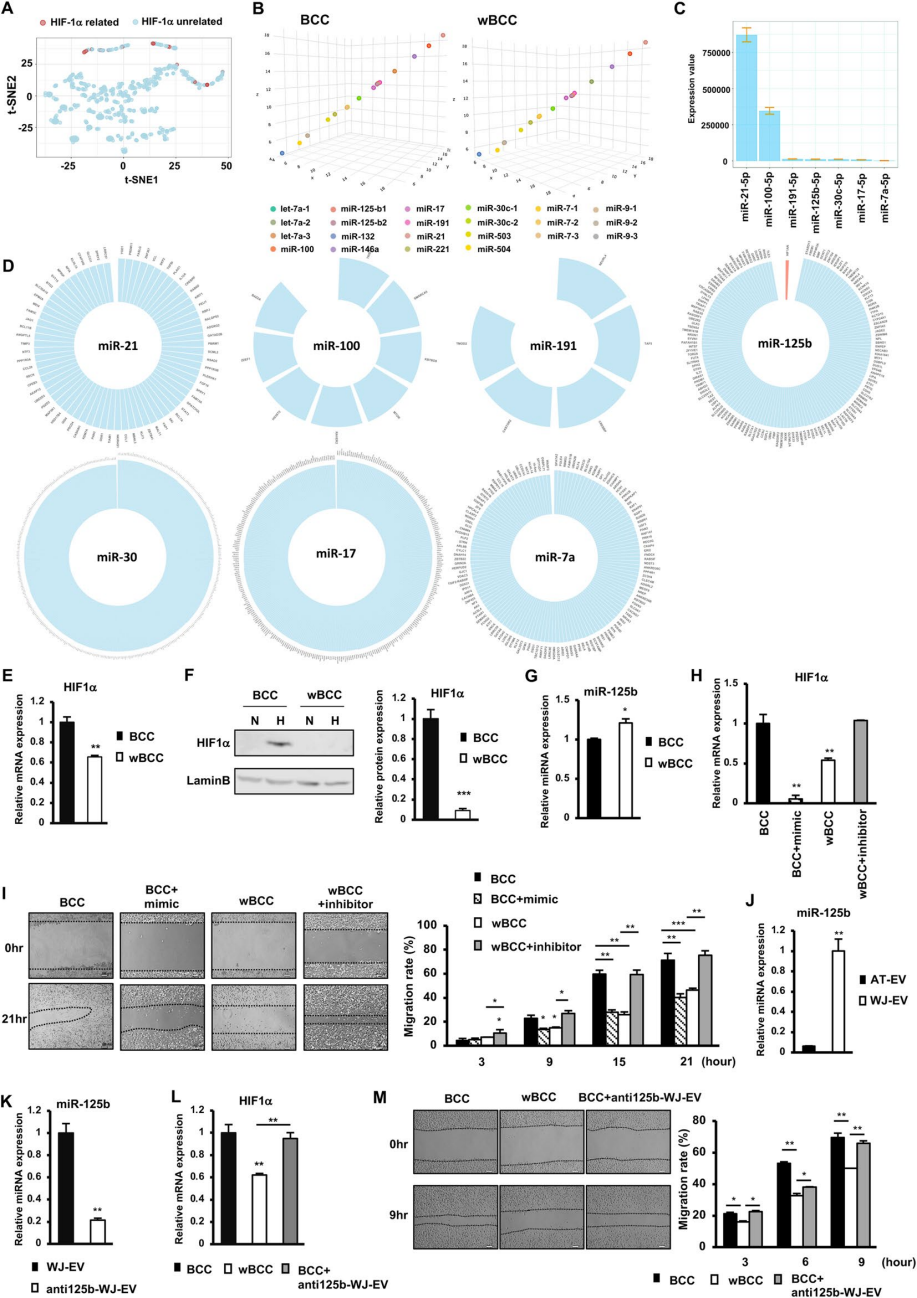
miRNA-125b from WJ-EV was involved the downregulation of the expression of HIF1α in wBCC. (**A**) The miRNAs related to the expression of HIF1α were analyzed by t-Distributed Stochastic Neighbor Embedding (t-SNE). (**B**) The expression of miRNAs related to the HIF1α expression in BCC and wBCC was presented in a 3-dimensional plot. (**C**) The expression of miRNAs related to HIF1α in BCC and wBCC. (**D**) Screening results of target genes which directly regulated by miRNAs related to HIF1α in BCC and wBCC, from a miRNA database, miRDB (http://mirdb.org/). Red color indicate anti-HIF1α (HIF1α-AN). A zoom-in image of target genes were shown in Supplementary Fig. S5. (**E**) HIF1α gene expression of BCC and wBCC, n = 6, **p < 0.01. (**F**) HIF1α protein expression in BCC and wBCC, n = 3, ***p < 0.001. N: normoxia, H: hypoxia. Full-length blots were shown in Supplementary Fig. S6. (**G**) The expression of miR-125b in BCC and wBCC, n = 3, *p < 0.05. (**H**) The gene expression of HIF1α in BCC, BCC with the overexpression of miR125b (treated with 50 nM of mimics of miR125b), wBCC, and wBCC with the inhibition of miR125b (treated with 25 nM of inhibitor of miR125b), n = 6, **p < 0.01. (**I**) The migration assay of BCC, BCC with overexpression of miR125b (treated with 50 nM of mimics of miR125b), wBCC, and wBCC with the inhibition of miR125b (treated with 25 nM of miR125b inhibitor), n = 3, *p < 0.05, **p < 0.01, ***p < 0.001. (**J**) The expression of miR-125b in WJ-EV and AT-EV, n = 3, **p < 0.01. (**K**) The expression of miR-125b in WJ-EV and WJ-EV-derived from WJ-MSC treated with 50 nM of miR-125b inhibitor (anti125b-WJ-EV), n = 3, **p < 0.01. (**L**) The expression of HIF1α in BCC, wBCC and BCC-internalized with anti125b-WJ-EV, n = 3, **p < 0.01. (**M**) The migration of BCC, wBCC and BCC-internalized with anti125b-WJ-EV, n = 3, *p < 0.05, **p < 0.01.

HIF1α is a crucial factor that regulates cancer cell behavior under hypoxic conditions in the tumor microenvironment. Therefore, we next focused on miRNAs that are related to the regulation of the HIF1α signaling pathway. A preprocessing procedure in the IPA showed 14 miRNAs (mir-21, mir-100, mir-191, mir-125b, mir-30c, mir-17, mir-7a, let-7a, mir-221, mir-146a, mir-503, mir-9, mir-504, and mir-132) were involved in the HIF1α signaling pathway. These miRNAs were also verified by a t-SNE analysis. As shown in Fig. 5A, the cluster of miRNAs related to the HIF1α signaling pathway suggested that these 14 miRNAs may have similar functions in regulating the HIF1α signaling pathway. Interestingly, a 3-dimensional plot showed that these 14 miRNAs were differentially distributed, suggesting that they have different expression patterns in BCC and wBCC (Fig. 5B). Among these 14 miRNAs, 7 miRNAs were found to be highly expressed in both BCC and wBCC (Fig. 5C). Next, we screened the target genes (target score > 90) of these 7 miRNAs in a miRNA target prediction and functional annotation database, miRDB (http://mirdb.org/)^38^. The results showed that only miR-125b directly regulates HIF1α inhibitor (Fig. 5D).

In addition, to determine the role of the HIF1α signaling pathway in wBCC, we compared the expression of HIF1α in wBCC and BCC under hypoxic conditions. As a result, under hypoxic conditions, wBCC showed the impaired expression of HIF1α, at both the mRNA level (Fig. 5E) and the protein level (Fig. 5F), in comparison to BCC. In addition, wBCC showed the higher expression of miR-125b (*p* < 0.05, Fig. 5G), in comparison to BCC. Moreover, to determine the role of miR-125b in the regulation of the expression of HIF1α, we overexpressed miR-125b in BCC. The overexpression of miR-125b significantly inhibited the expression of HIF1α in BCC (Fig. 5H). Of note, treatment of wBCC with an miR-125b inhibitor significantly reversed the downregulation of HIF1α; the expression of HIF1α was induced in wBCC treated with miR-125b inhibitor (2.1-fold increase, *p* < 0.001, Fig. 5H), suggesting that miR-125b is a direct inhibitor of the expression of HIF1α in wBCC.

We next examined the effects of miR-125b on the migratory ability of BCC and wBCC. As shown in Fig. 5H, in comparison to BCC, BCC with the overexpression of miR-125b showed a significantly impaired migratory ability, which was similar to the ability of wBCC. In addition, the treatment of wBCC with an miR-125b inhibitor significantly induced the migratory ability of these cells to a level that was similar to the ability of BCC (Fig. 5I), suggesting that miR-125b is the key factor in the impairment of the migratory ability of wBCC.

Several studies suggested that MSC-EV modulate the cellular behavior of recipient cells by transferring their cargo, which contains miRNAs^15,16^. Therefore, we next examined the expression of miR-125b in the cargo of WJ-EV and AT-EV to determine the possibility that WJ-EV might transfer miR-125b to BCC, to generate the transformation of the wBCC phenotype. As expected, in comparison to AT-EV, WJ-EV showed significantly higher miR-125b expression levels (16.7-fold higher, *p* < 0.01, Fig. 5J). In order to examine the role of miR-125b derived from WJ-EV in the regulation of BCC, the silencing miR-125b expression in WJ-MSC was conducted, then EV were harvested (anti125b-WJ-EV). As a result, anti125b-WJ-EV showed the lower expression of miR125b, in comparison to WJ-EV (Fig. 5K). Next, anti125b-WJ-EV was internalized to BCC and the effects on HIF1α expression and migration were examined. The results showed that in contrast to wBCC, BCC-internalized with anti125b-WJ-EV showed no downregulation of HIF1α (Fig. 5L) and no impaired migration (Fig. 5M), in comparison to BCC. Therefore, these data suggested that miR-125b derived from WJ-EV is a key factor which impairs the expression of HIF1α and migration ability of wBCC.

Taken together, these data suggested that the transformed behaviors of wBCC might be involved in the impaired hypoxic response due to the downregulation of HIF1α. Of note, miR-125b was determined to be a key factor that inhibits the expression of HIF1α in wBCC and which might be transferred from WJ-EV to target cells.

## Discussion

**Figure 6:**
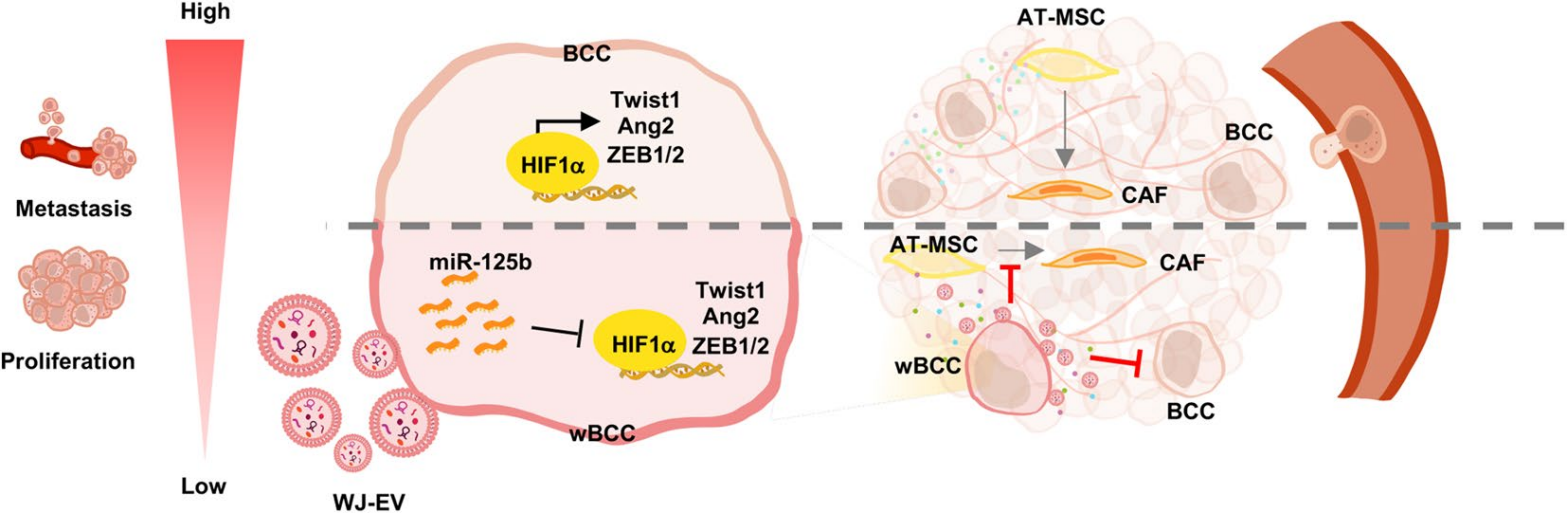
wBCC attenuated the development and metastasis of BCC. The incorporation of WJ-EV to BCC resulted in wBCC that showed the impaired expression of HIF1α and its target genes, which are involved in attenuating proliferation, metastasis and angiogenesis. wBCC and their derived EV attenuated cancer progression by reducing the proliferation and metastasis of the original BCC, the angiogenic abilities of EC and EPC, and the differentiation of AT-MSC to CAF.

In the present study, we found that the internalization of WJ-EV, but not AT-EV, resulted in the generation of wBCC with transformed phenotypes and behaviors that impaired the functions of several types of cells involved in the tumor microenvironment, including cancer cells, EC, EPC, and MSC. In addition to the transformed phenotypes of BCC, wBCC produced modified EV that had inhibitory effects on the functions of the original BCC, EC, and EPC in metastasis. Of note, the transformed behaviors of wBCC was involved in impairment of the expression of HIF1α by miR-125b, which might be transferred from WJ-EV after internalization (Fig. 6).

How EV modulate the cellular behaviors and functions of cancer cells in tumor progression under hypoxic conditions has remained unclear. A previous study showed that WJ-EV contain plenty of functional miRNAs, including the let7 family, miR122, and miR125b, which might regulate numerous signaling pathways in recipient cells^39^. In our study, we found that the internalization of WJ-EV to BCC resulted in wBCC with an altered miRNA profile. Interestingly, numerous miRNAs, which were upregulated in wBCC, have been reported as suppressors of tumor growth and metastasis of TNBC, including mir-200b and mir-148a^32,33^. In addition, by cross-validating the results of featured selection from LASSO regression with the results of a volcano plot, mir-148a, which was upregulated in wBCC, was suggested to be a promising key factor in wBCC. As mir-148a was reported to alleviate tumor metastasis in TNBC, it is suggested that it may be involved in the downregulation of signaling pathways that are related to the impaired metastatic ability of wBCC^33^.

HIF1α plays a crucial role in providing a favorable environment for tumor growth and metastasis^40^. A previous study showed that in comparison to the tumors in non-TNBC patients, the expression of HIF-1α is higher in tumors in TNBC patients^41,42^. Therefore, targeting the HIF1α signaling pathway has been considered to be an important treatment strategy for TNBC. In the present study, we suggested that WJ-EV as an effective tool for the downregulation of the HIF1α signaling pathway in BCC under hypoxic conditions. Our study suggested that WJ-EV transformed the phenotypes of wBCC by transferring miR-125b to BCC, which resulted in the impaired expression of HIF1α. In addition, our finding also highlighted the role of miR-125b/HIF1α signaling pathways in the impaired tumorigenic and metastatic abilities of wBCC. HIF1α was identified as a transcription factor of numerous genes related to the EMT, stem-like properties and metastasis of tumor cells, including Twist1, ZEB1 and ZEB2^23,24,43^. Consistent with the downregulation of these target genes of HIF1α, wBCC showed impaired in vivo tumorigenic ability in the tumor xenograft mouse model and metastatic ability in the lung metastasis mouse model.

Furthermore, our small RNA sequencing results suggested that in addition to miR-125b, other miRNAs involved in HIF1α signaling pathways, including miR-21, miR-100, miR-191, miR-30c, miR-17 and miR-7a, highly expressed in wBCC and BCC. In order to get an overview of the roles of these miRNAs in the regulation of HIF1α, we performed a systematic review using three databases: PUBMED (https://pubmed.ncbi.nlm.nih.gov/), EMBASE (https://www.embase.com/) and Web of Science (https://www.webofscience.com/wos/woscc/basic-search) using keyword (microRNA OR miRNA OR miR) AND ('21' OR '100' OR '191' OR '125b' OR '7a') AND (HIF1a OR HIF1α OR HIF1alpha) (Supplementary Fig. S2). This search was completed on May 30th, 2022. As a result, from 821 potentially relevant articles identified through the search, the direct inhibitory effect on HIF1a expression of miR-125b was reported in six articles, of miR-100 was reported in two articles, and of miR-21 was reported in one article. No report of the direct inhibitory effect on HIF1α expression of miR-191, miR-30c, miR-17, miR-71 was found (Supplementary Table S1). The systematic review result showed that it is noteworthy for further studies to examine the role of miR-100 and miR-21 in wBCC.

Of note, our results showed that besides the reduced tumorigenic abilities, wBCC possessed paracrine effects, which impaired the tumorigenic and metastatic abilities of the original BCC, suggesting that internalization with WJ-EV also transformed the cancer cells. Recent studies suggested that the modification of cancer cell behavior, to interrupt the original homeostasis and regulate the tumor development, is a promising future cancer treatment^44,45^. Interestingly, in addition to the inhibitory effects on the original BCC, our data suggested that wBCC also showed transformed effects, which impaired the ability of numerous types of cells to support tumor progression. In particular, wBCC impaired the angiogenic abilities of EC and EPC. EC and EPC are key players in angiogenesis and neovascularization, which form new blood vessels in the tumor vascular network to supply oxygen and nutrients, and generate the route for metastasis of tumor cells. Therefore, it is suggested that the level of angiogenesis reflects the aggressiveness of a tumor. Indeed, several studies reported that the tumors of patients with TNBC show higher angiogenic abilities in comparison to patients with non-TNBC^46,47^; thus, anti-angiogenesis is important for the treatment of aggressive cancers, such as TNBC.

In addition to the inhibitory effects on the tumorigenesis of BCC and angiogenesis, wBCC impaired the ability of AT-MSC to differentiate to CAF. CAF is one of the largest heterogeneous cell populations in the tumor microenvironment, and has diverse functions that support tumor progression and metastasis. A previous study reported that in response to the signals from BCC, AT-MSC differentiate to CAF, which promote the invasive ability of BCC^48^. Of note, our data showed that CAF generated from AT-MSC in response to BCC enhanced, while those generated from AT-MSC in response to wBCC impaired the in vivo tumorigenesis of BCC. Therefore, in comparison to BCC, wBCC showed contrasting cellular behavior and communication that attenuated the development of breast tumors. For the clinical application, further studies should be undertaken to examine whether the inhibitory effects of wBCC on tumorigenesis are temporary or permanent.

In the primary tumor, cancer cell-derived EV play a vital role in intercellular communication to support tumor growth and metastasis^5,18,49^. Interestingly, in our study, treatment with WJ-EV not only altered the behavior of BCC but also induced the recipient cells to produce modified EV. wBCC-EV reflected the ability of the parental wBCC, which showed that the functions were transformed to attenuate the tumor progression, for example by reducing cell proliferation, sphere formation, and in vivo metastasis of BCC and the angiogenic abilities of EC and EPC^6,26^. Our data were consistent with several previous studies that showed that the secretome of cancer cells treated by MSC-EV inhibits the angiogenic and migration ability of EC^15,16^. Moreover, in consideration of the fact that tumors consist of numerous types of cells, further studies should be undertaken to investigate the effects of wBCC-EV on other types of cells in the tumor microenvironment and on the in vivo functions of wBCC-EV in the process of tumorigenesis.

## Conclusion

In summary, our results suggested that the internalization of WJ-EV to BCC impaired the tumorigenic abilities of BCC, which might be involved in the transfer of miRNA-125b to downregulate the HIF1α signaling pathway. Of note, the recipient BCC, considered to be a wBCC, showed transformed behaviors. These cells might reside in tumors, interrupt the intercellular communication, and regulate the functions of the surrounding cells to create an unfavorable tumor microenvironment that attenuates tumorigenesis and metastasis in TNBC. Therefore, WJ-EV may have a significant inhibitory effect on the process of tumor formation, and therefore lead to the development of new cancer therapies along with other cancer treatment methods.

## Materials and methods

### Ethics statement

All experiments were conducted according to the amended Declaration of Helsinki and followed the recommendations in the ARRIVE guidelines. In addition, all experiments were approved by the Ethics Committee of the University of Tsukuba. The collection of human samples was performed with the informed consent of the donors. The in vivo experiments were approved by the Animal Care Committee of the University of Tsukuba.

### Cell isolation and culture

To isolate MSC from Wharton’s Jelly, human umbilical cord was obtained from the Department of Obstetrics and Gynecology, University of Tsukuba Hospital, Japan (n = 4, female, average age 32 years). Human umbilical cords were cut to expose the blood vessels and Wharton’s jelly. Then, Wharton’s jelly was collected and cut into 1–2 mm pieces following by incubating with 0.1% Collagenase solution at 37 °C for 30 min. After incubation, the solution was centrifuged at 1600 rpm for 7 min, then washed with PBS. The pellet was then carefully plated on a culture dish containing MSC culture medium (Iscove’s modified Dulbecco’s medium (IMDM) (Thermo Fisher Scientific, Carlsbad, CA, US) with 10% FBS, 2 mg/mL l-glutamine (Thermo Fisher Scientific), 5 ng/mL human basic-FGF (Peprotech, London, UK), and 0.1% (v/v) penicillin–streptomycin (100 U/mL penicillin, 0.1 mg/mL streptomycin; Thermo Fisher Scientific) at 37ºC in 5% CO_2_ and a humidified atmosphere for 5 days. Frozen cell stocks were prepared using Cell Banker solution (ZENOAQ, Koiyama, Japan) and stored in liquid nitrogen for further experiments.

To isolate MSC from adipose tissues, human adipose tissue was obtained from the Department of Cardiovascular Surgery, University of Tsukuba Hospital, Japan (female, n = 4; average age, 80 years; HbA1c < 6). The adipose tissue was minced and digested with 0.1% collagenase (Invitrogen, Waltham, Massachusetts, US) in PBS at 37 °C for 1 h followed by centrifugation to harvest cell pellets and cultured in culture medium of IMDM (Invitrogen) supplemented with 10% heat-inactivated FBS (Invitrogen), 2 mg/mL l-glutamine (Invitrogen), 100 U/mL penicillin (Invitrogen), and 5 ng/mL bFGF (Peprotech, Rocky Hill, NJ). Cells were maintained and cultured at 37 °C in a 5% CO_2_ atmosphere.

To culture BCC, the MDAMB-231 cell line was purchased from the American Type Culture Collection (ATCC, Manassas, VA, US), and cultured in IMDM medium, with 1% Penicillin–Streptomycin, 5% FBS and 2 mg/mL l-glutamine. In addition, EPC were isolated from umbilical cord blood, as previously described^50^ and cultured in IMDM medium, with 1% Pen-Strep, 10% FBS, 2 mg/mL l-glutamin and 5 ng/mL bFGF. For EC culture, human Umbilical Vein Endothelial Cell (HUVEC) was purchase from ATCC and cultured in Endothelial Cell Growth Medium (PromoCell, Heidelberg, DE, Germany), with 1% Penicillin–Streptomycin.

### Isolation of MSC-derived extracellular vesicles (MSC-EV) and conditional medium collection

The conditioned medium from MSC culture was collected and centrifuged at 1000×*g* for 5 min, then at 2100×*g* for 20 min, and ultracentrifuged at 100,000×*g* for 70 min at 4 °C (Optima L-100 K, Beckman Coulter, Brea, CA, US). The pellets were stained with PKH26 (PKH26 linker, Lot.MKCG0926, SIGMA, Burlington, MA, US) for 5 min at room temperature. After washing by PBS, EV were collected. The total protein of EV was measured by the Bradford method (BioRad, CA, USA), the size of EV was measured by a Particle Size Analyzer (FDLS3000; Shimadzu Corporation, Kyoto, Japan), and the morphology was analyzed by transmission electron microscopy (JEM-1400Flash, JEOL Ltd., Tokyo, Japan).

### Internalization of BCC with MSC-EV

BCC were treated with WJ-EV or AT-EV at a concentration of 75 ng EV/10^4^ cells. After 24 h, BCC was continuously treated with a second dose of WJ-EV or AT-EV, at a concentration of 75 ng EV/10^4^ cells.

### Cell migration assay

Cells were seeded into the 24-well cell culture dish and incubated under 37 °C in 1% O_2_ and 5% CO_2_ for 24 h to reach 100% confluence. Cells were treated with mitomycin C for 2 h under 37 °C in 1% O_2_ and 5% CO_2_, then a scratch was made and washed with PBS three times. The gap of cells was observed every 6 h under a microscope (BZ-X710, Keyence, Osaka, Japan).

### Gene expression analysis

Total RNA was isolated using Sepasol-RNA I Super G (Nacalai Tesque, Kyoto, Japan), according to the manufacturer’s protocol. Total RNA (1 μg) was reverse transcribed using a RT-PCR kit (TOYOBO, Osaka, Japan). cDNA was analyzed using a GeneAmp 7500Fast Real-Time PCR System (Applied Biosystems, Waltham, MA, US) using SYBR green reagent (TOYOBO) with the primer sets shown in Table 1. The expression levels of the target genes were analyzed using the ΔΔCt method.

**Table 1:** Primers used for quantitative polymerase chain reaction.

Gene name		Sequence
β-actin	F	GTGCGTGACATTAAGGAGAAGCTGTGC
	R	GTACTTGCGCTCAGGAGGAGCAATGAT
HIF1α	F	TGCTCATCAGTTGCCACTTC
	R	AAAACATTGCGACCACCTTC
CDK2	F	CTCTTCCCCTCATCAAGAGCTATC
	R	CTCATGGTGTAAGTACGAACAGG
CDK4	F	AGCCGAAACGATCAAGGATCTG
	R	GTTCCACCACTTGTCACCAGAATG
CDK6	F	GATCTCTGGAGTGTTGGCTGCATA
	R	TTCTTCTCCTGGGAGTCCAATCAC
ZEB1	F	CAGCTCTGGGTGGAGAAGAC
	R	CCTGACCCACTTCCAACAGT
ZEB2	F	CTGCTTGGCAAGGGTAAGAG
	R	TTTAAGGCCAGCTCAAGCAT
Twist1	F	AGCCGCAGAGACCTAAACAA
	R	CACGCCCTGTTTCTTTGAAT
Bcl2	F	CTGCGAAGAACCTTGTGTGA
	R	TGTCCCTACCAACCAGAAGG
Ang1	F	AATGAGTTTATTTTTGCCATTACCA
	R	CCCAGTGTGACCTTTTAAATACAAC
Ang2	F	CACACACCACCTGAAAATGC
	R	TTGGAGTTTTGGCTGCTCTT
TGF-β	F	AGAGCTCCGAGAAGCGGTACCTGAACCC
	R	GTTGATGTCCACTTGCAGTGTGTTATCC
bFGF	F	GATCGAGCTCACTGTGGAGT
	R	CAGAGTGTTGCTGTGACCAG
IL6	F	ACAAGAGTAACATGTGTGAAAGCAG
	R	TATACCTCAAACTCCAAAAGACCAG
CXCR4	F	GGTGGTCTATGTTGGCGTCT
	R	TGGAGTGTGACAGCTTGGAG
TNFα	F	TCCTTCAGACACCCTCAACC
	R	AGGCCCCAGTTTGAATTCTT
BAX	F	TCTGACGGCAACTTCAACTG
	R	TCTCTCTCCATGCCCTCTGT
Survivin	F	CCGAGCTCCAGAAGTGACTC
	R	GGGCCACTACCGTGATAAGA
FAP	F	CTCTTCCCCTCATCAAGAGCTATC
	R	CTCATGGTGTAAGTACGAACAGG
FSP	F	AGCCGAAACGATCAAGGATCTG
	R	GTTCCACCACTTGTCACCAGAATG
Vimentin	F	CAGCTCTGGGTGGAGAAGAC
	R	CCTGACCCACTTCCAACAGT
RNU48		GATGACCCCAGGTAACTCTGAGTGTGTCGCTGATGCCATCACCGCAGCGCTCTGACC
miR-125b		UCCCUGAGACCCUAACUUGUGA
miR-125b mimics		UCCCUGAGACCCUAACUUGUGA
miR-125b inhibitor		UCCCUGAGACCCUAACUUGUGA

### Mammosphere assay

BCC were seeded in a 6-well cell culture plate with an ultra-low attachment surface (Corning, New York, US) at a density of 1000 cells/cm^2^, and treated with mammosphere medium (Stemcell technologies, Vancouver, Canada). Cells were cultured at 37ºC under 1% O_2_ for 5 days. The cell clusters were observed and counted under a microscope.

### CAF differentiation

Adipose tissue-derived MSC (1 × 10^5^ cells/well) were seeded in a 24-well cell culture dish and cultured at 37 °C under 5% CO_2_ overnight. The 3.0 µm Transwells (Corning) containing a number of 1 × 10^5^ cells/well BCC or wBCC were placed in each wells in 24-well cell culture dish. The Transwell coculture system sets were cultured at 37ºC under 1% O_2_ for 14 days.

The differentiation of CAF was examined by immunostaining with Rabbit anti-Vimentin as the primary antibody (1:500, ab137321, abcam, Cambridge, UK) and Goat anti-Rabbit IgG as the second antibody, and Dapi (1:500, ab150077, abcam, Dapi 1:1000) then observing under under a microscope (Keyence, BZ-X710).

### Tube formation assay

The 4-well cell culture plate (Thermo Fisher Scientific) was coated with 250 µL of Matrigel (Corning) in each well. EC (1 × 10^5^) and EPC were seeded with 500 µL culture medium or conditional medium in each well. Pictures were taken every three hours.

### Western blotting

Total protein was extracted from BCC pellets according to a previously described method^50^. Protein (30 μg) was used for electrophoresis with SDS–polyacrylamide gels and transferred onto PVDF membranes (Merck Millipore, Burlington, MS, US). After blocking, the membranes were cut and incubated with specific primary antibody, such as rabbit anti-human CDK4 antibody (GTX10299, GeneTex, Irvine, CA, US) or rabbit anti-human CDK6 antibody (GTX10299, GeneTex), at 1:1000 dilution at 4 °C overnight. Rabbit anti-β-actin (GTX109639, Genetex) at 1:1000 dilution was used as the loading control. In order to examine the expression of HIF1α, nuclear protein was extracted and applied for the electrophoresis, following by transferring to the membranes. The membranes were blocking, cut and incubated with with rabbit anti-human HIF1α antibody (NB100-479, Novus Biologicals, CO, US) or goat anti-Lamin B antibody (sc-6217, Santa Cruz Biotechnology, Dallas, TX, US) at 1:1000 dilution at 4 °C overnight.

For EV markers, the membranes were incubated with rabbit anti-CD63 (CSB-PA006039, Cusabio Tachnology LLC, Houston, TX, US), rabbit anti-TSG101 (CSB-PA060017, Cusabio Technology LLC), or goat anti-β-actin (sc-47778, Santa Cruz Biotechnology) at 1:1000 dilution. The membrane was then washed and incubated with the following secondary antibodies: horseradish peroxidase (HRP)-conjugated rabbit anti-goat IgG (Invitrogen) and HRP-conjugated goat anti-rabbit IgG (Invitrogen) at 1:10,000 dilution at room temperature for 2 h, then incubated with chemiluminescence HRP substrate (EMD Millipore, Burlington, MA, US) for 1 min. The protein expression was detected using an Image Quant LAS 4000 System (GE Healthcare, Chicago, IL, US).

### In vivo tumor growth assay

Female nude mice were purchased from Charles River Japan, Inc. (Yokohama, Japan), maintained on a 12-h light/dark cycle with ad libitum access to food and water until eight weeks of age. Breast cancer cells (10^6^) were collected, and re-suspended in 100 µL of PBS and 100 µL of Matrigel (Corning) for subcutaneous injection. The tumor size was measured. Mice that received WJ-EV treatment were injected with 10 µg of WJ-EV in 30 µL of PBS at the tumor sites two times per week.

### In vivo metastatic assay

Female C57BL/6 mice were purchased from Charles River Japan, Inc., maintained on a 12-h light/dark cycle with ad libitum access to food and water until eight weeks of age. Breast cancer cells (10^5^) were collected, and re-suspended in 200 µL of PBS for tail-vein injection. As immunosuppression, the mice received cyclosporin A (20 mg/kg; Wako) every day for two weeks, then, lung tissues were collected and fixed in 4% paraformaldehyde, embedded in O.C.T compound (Sakura Finetek, Tokyo, Japan), frozen in liquid nitrogen. Lung samples were then sectioned and stained with hematoxylin and eosin (H&E) to identify metastatic cancer cells.

### Small RNA sequencing analysis

The small RNA sequencing was performed according to the methods described in a previous report^51^. Briefly, cDNA was synthesized by using 500 ng of total RNA extracted from BCC or wBCC then applied for sequencing using an Illumina NextSeq500 (Illumina, San Diego, CA, USA). The NEBNext Small RNA Library Prep Kit (E7330, New England Biolabs, Ipswich, MA, USA) was used to build a sequencing small RNA library, which was selected according to size using AMPure beads (NC9933872, Thermo Fisher Scientific, Inc) and verified using a Bioanalyzer DNA High-sensitivity Kit (5067-4626, Agilent). Reads were grouped by sequence and analyzed using a small RNA analysis tool and CLC Genomics Workbench (Ver.9.5.3, Qiagen, Germantown, MD, USA) then matched to miR-base annotated microRNAs (miRbase v21). A total count of one million or z-score normalization was used to normalize the raw counts of each sample.

A differential expression analysis was performed using the same gene count matrix. The packages, edgeR (v.3.28.1), pheatmap (v.1.0.12), and Rtsne (v.0.15) were applied according to the procedures described in the package documentation. For each method, comparisons were performed among three replicates from BCC samples, with three replicates from wBCC samples. The significant miRNAs in BCC and wBCC were selected by the LASSO regression algorithm (glmnet v.4.1-3). Denote ${x}_{i}$ as a sample and ${y}_{i}$ as the label of sample ${x}_{i}$. ${y}_{i}$ is set as 1 if ${x}_{i}$ belongs to if wBCC and set as 0 if ${x}_{i}$ belongs to BCC. The significance of each miRNA is calculated based on ${w}_{j}$ by minimizing the following problem.

$$ \sum\limits_{i}^{n} {\left( {y_{i} - \sum\limits_{j} {x_{ij} w_{j} } } \right)^{2} + \lambda \sum\limits_{j}^{p} {\left| {w_{j} } \right|} } , $$

where $\lambda$ is a tuning parameter to control the strength of the L1 penalty.

### Analysis of miRNA gene expression

Total RNA of cell samples were isolated by Sepasol-RNA I Super G (Nacalai Tesque) in accordance with the manufacturer’s protocol and the total RNA of EV samples were isolated using ISOGEN-LS (Nippon gene, Tokyo, Japan) in accordance with the manufacturer’s protocol. Total RNA (1 µg) was reverse transcribed using a TaqMan® MicroRNA Reverse Transcription Kit (Applied Biosystems). cDNA (500 ng) was analyzed using a GeneAmp 7500Fast Real-Time PCR System (Applied Biosystems) using TaqMan 2 × Universal PCR Master Mix, with AmpErase UNG (Applied Biosystems). The RNU48 was used as internal control (ThermoFisher). The expression levels of the target genes were analyzed using the ΔΔCt method. The sequences of the primer sets used for PCR are shown in Table 1.

### Overexpression and inhibition of miRNA

BCC (3 × 10^4^) were seeded with 1 mL of culture medium in a 24-well cell culture dish, and were cultured under hypoxic conditions. After 48 h, the medium in each well was replaced with fresh medium. Lipofectamine (Lipofectamine RNAiMAX reagent, Invitrogen) was diluted in Opti-MEM I Reduced Serum Media (Thermo Fisher Scientific, Inc.), then mixed with the miR125b inhibitor (Lot. WDAD5014, SIGMA) or miR125b mimics (Lot. WDAA6220, SIGMA), which were separately diluted in Opti-MEM I Reduced Serum Media. The mixture of miR125b mimics or miR125b inhibitor was separately added to each well. The cells were incubated under hypoxic conditions for 48 h, then the gene expression and migration were assayed.

### Statistical analysis

Data were analyzed by the Mann Whitney U-test. Results are presented as the mean ± standard deviation. P values of < 0.05 were considered to indicate statistical significance. All analyses were performed using the GraphPad Prism 5 software program (GraphPad Software, San Diego, CA, US).

## Supplementary Information

Supplementary Information.

## Data Availability

The data in the present study are available from the corresponding author upon reasonable request. Small RNAseq data are deposited in Gene Expression Omnibus (GEO) database, National Center for Biotechnology Information (NCBI), with a GEO accession number GSE197863 (https://www.ncbi.nlm.nih.gov/geo/query/acc.cgi?acc=GSE197863) and token number: “engtumqoppijdmh”.
