# Supplementary material for: Extracellular vesicles derived from Wharton’s Jelly mesenchymal stem cells inhibit the tumor environment via the miR-125b/HIF1α signaling pathway
Source: Sci Rep. 2022 Aug 8;12:13550. doi: 10.1038/s41598-022-17767-y (PMC9359975; doi:10.1038/s41598-022-17767-y)
Supplement: Supplementary file 1 — Supplementary Information. [file 41598_2022_17767_MOESM1_ESM.docx]

**Extracellular Vesicles Derived from Wharton’s Jelly Mesenchymal Stem Cells Inhibit the Tumor Environment via the miR-125b/HIF1α Signaling Pathway**

Yun Hsuan Chang^1,2^, Vuong Cat Khanh^2^, Ngo Nhat Hoang^2^, Toshiharu Yamashita^2^, Xiucai Ye^3^, Yasunori Futamura^3^, Mizuho Fukushige^2^, Mana Obata-Yasuoka^4^, Hiromi Hamada^4^, Motoo Osaka^5^, Yuji Hiramatsu^5^, Tetsuya Sakurai^1,3^ and Osamu Ohneda^1,2^

^1^Ph.D. Program in Humanics, University of Tsukuba, 1-1-1 Tennodai, Tsukuba, Ibaraki 305-8575, Japan

^2^Graduate School of Comprehensive Human Science, Laboratory of Regenerative Medicine and Stem Cell Biology, University of Tsukuba, 1-1-1 Tennodai, Tsukuba, Ibaraki 305-8575, Japan

^3^Department of Computer Science, University of Tsukuba, 1-1-1 Tennodai, Tsukuba, Ibaraki 305-8575, Japan

^4^Department of Obstetrics and Gynecology, University of Tsukuba

^5^Department of Cardiovascular Surgery, University of Tsukuba

Address correspondence to: Osamu Ohneda, MD, PhD, Laboratory of Regenerative Medicine and Stem Cell Biology, University of Tsukuba, 1-1-1 Tsukuba 305-8575, Japan.

E-mail: oohneda@md.tsukuba.ac.jp


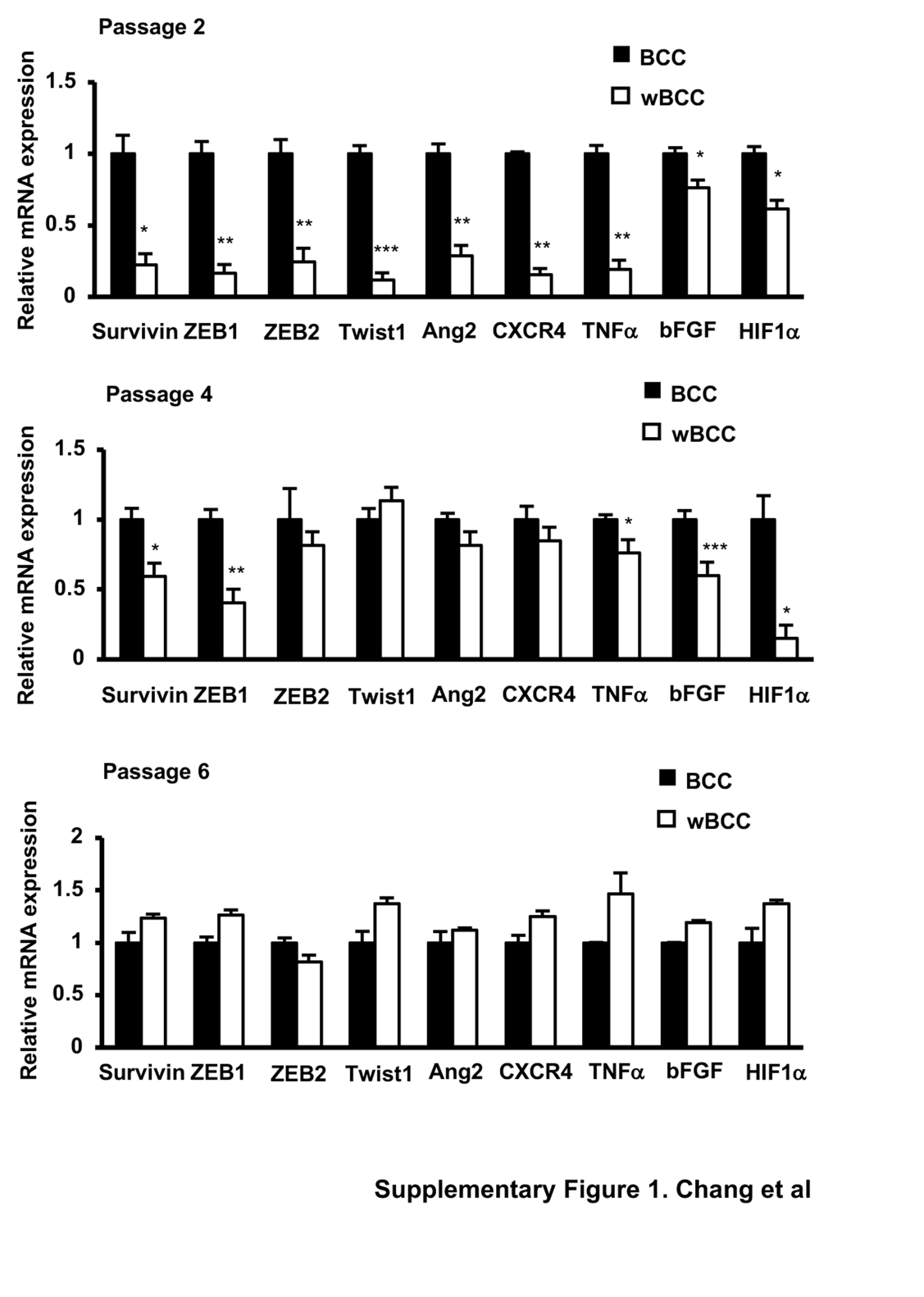


**Supplementary Figure 1.** Expression of genes related to EMT and migration in BCC and wBCC after being passaged in *in vitro* culture. n=3, *p<0.05, **p<0.01, ***p<0.001. Experiments were performed in triplicate.

**
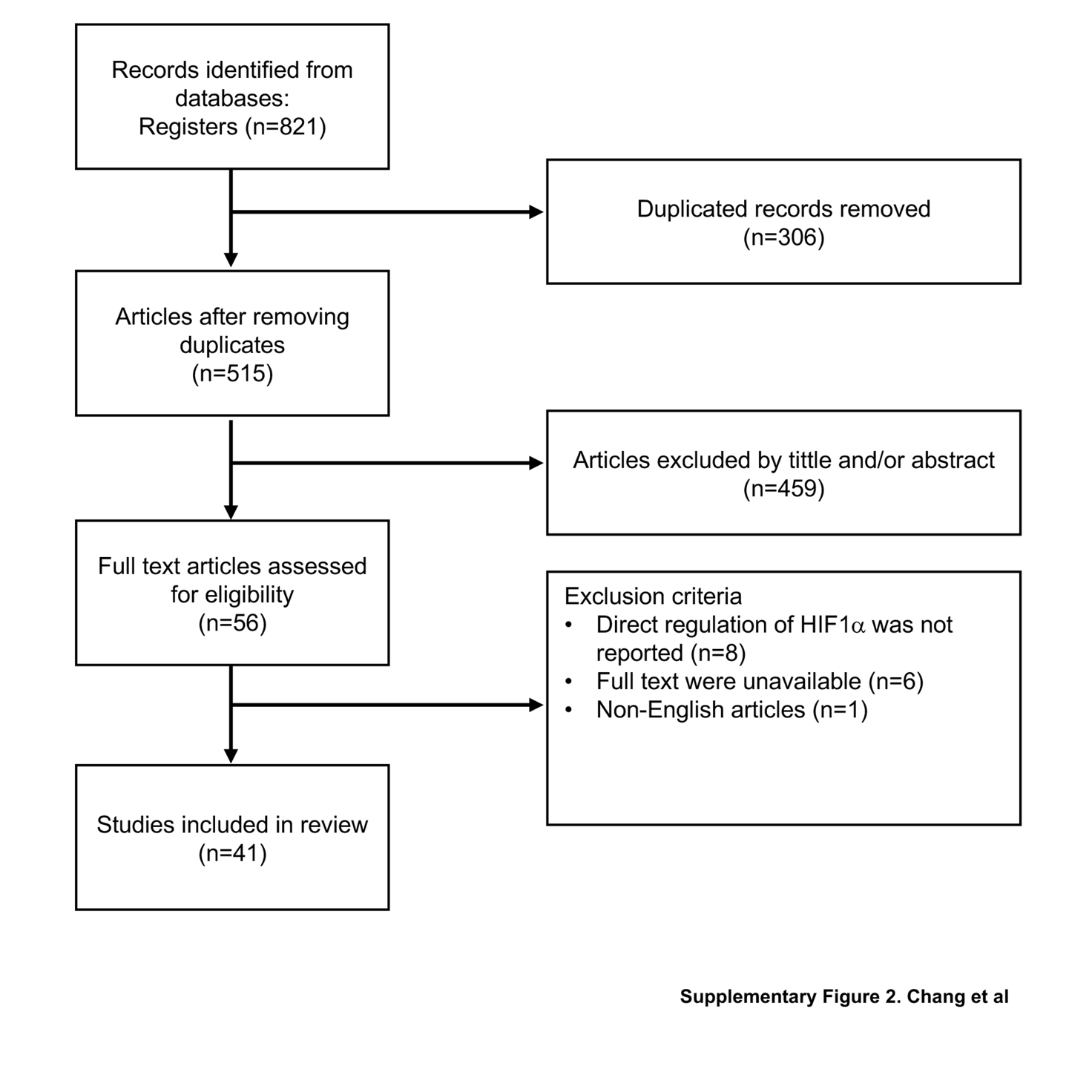
**

**Supplementary Figure 2.** Flow of systematic review. To investigate the miRNAs which directly inhibit the expression of HIF1α, a literature searching was conducted on three different databases: PubMed (https://pubmed.ncbi.nlm.nih.gov), Web of Science (https://www.webofscience.com/wos/woscc/basic-search) and EMBASE (https://www.embase.com) using keyword (microRNA OR miRNA OR miR) AND ('21' OR '100' OR '191' OR '125b' OR '7a') AND (HIF1a OR HIF1α OR HIF1alpha). This search was completed on May 30^th^, 2022. An article was included if it showed the experimental data in which miRNA of interest directly inhibited the expression of HIF1α. All the procedure of the systematic review and data extraction were conducted by two independent reviewers (N-H Ngo and C-K Vuong). The results of this systematic review were summarized in Supplementary Table 1.


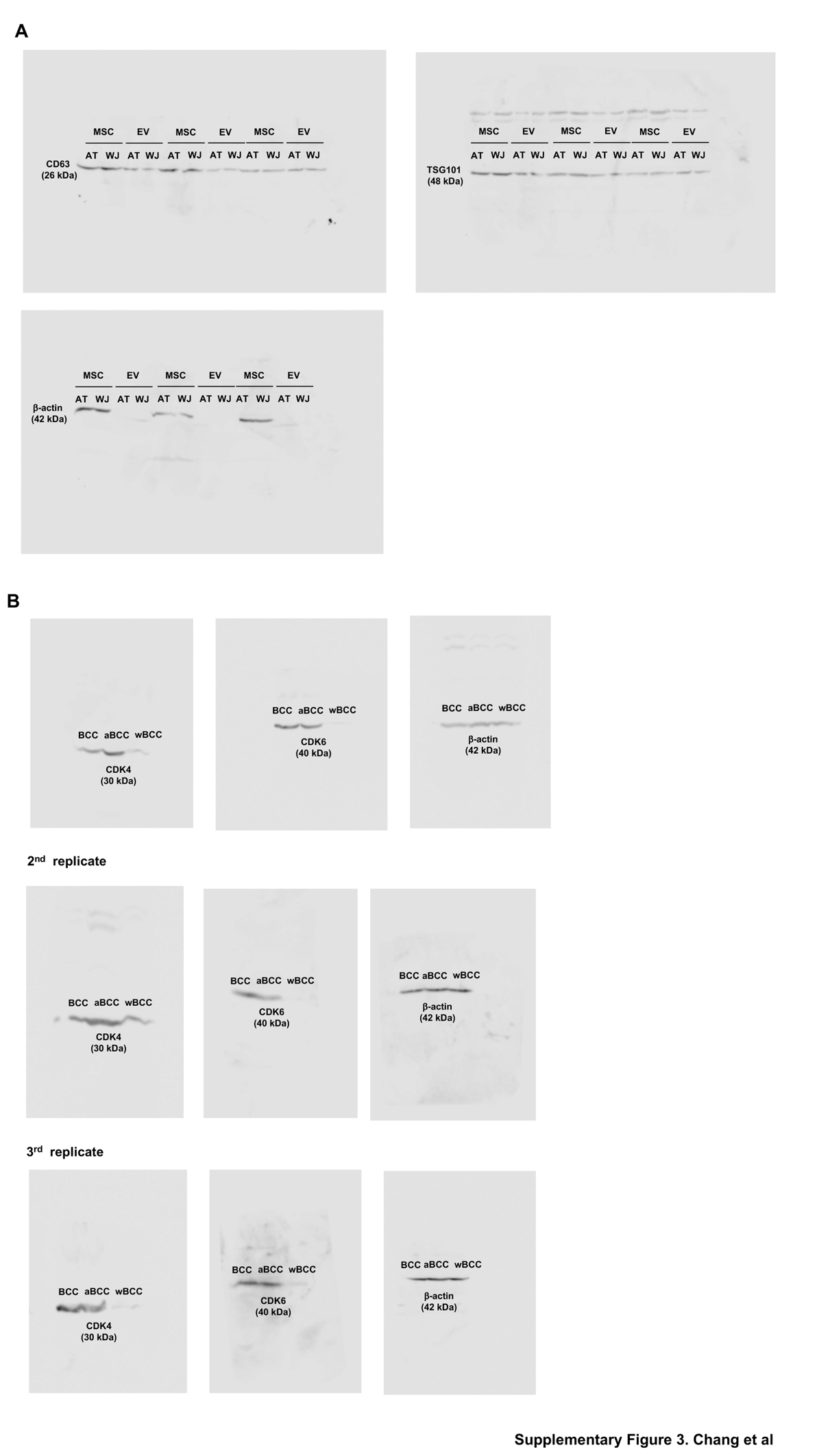


**Supplementary Figure 3. Full-length blots of Western blot images. A.** Markers of AT-EV and WJ-EV shown in Figure 1B. **B.** CDK4 and CDK6 protein expression shown in Figure 1G.


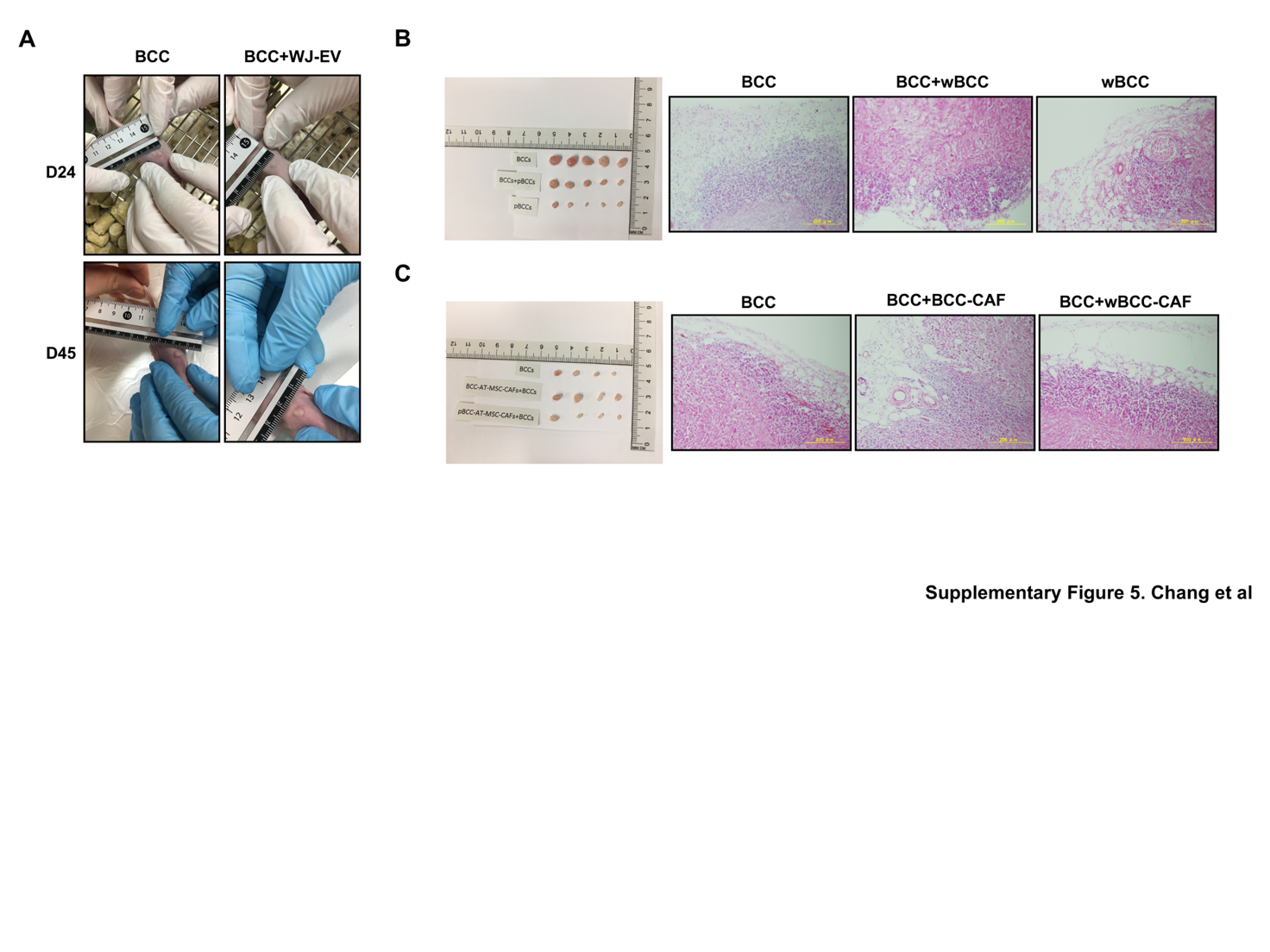


**Supplementary Figure 4. A.** Raw images of tumors in Figure 1L, **B.** Raw images of tumors and HE staining in Figure 2E, **C.** Raw images of tumors and HE staining in Figure 2M.


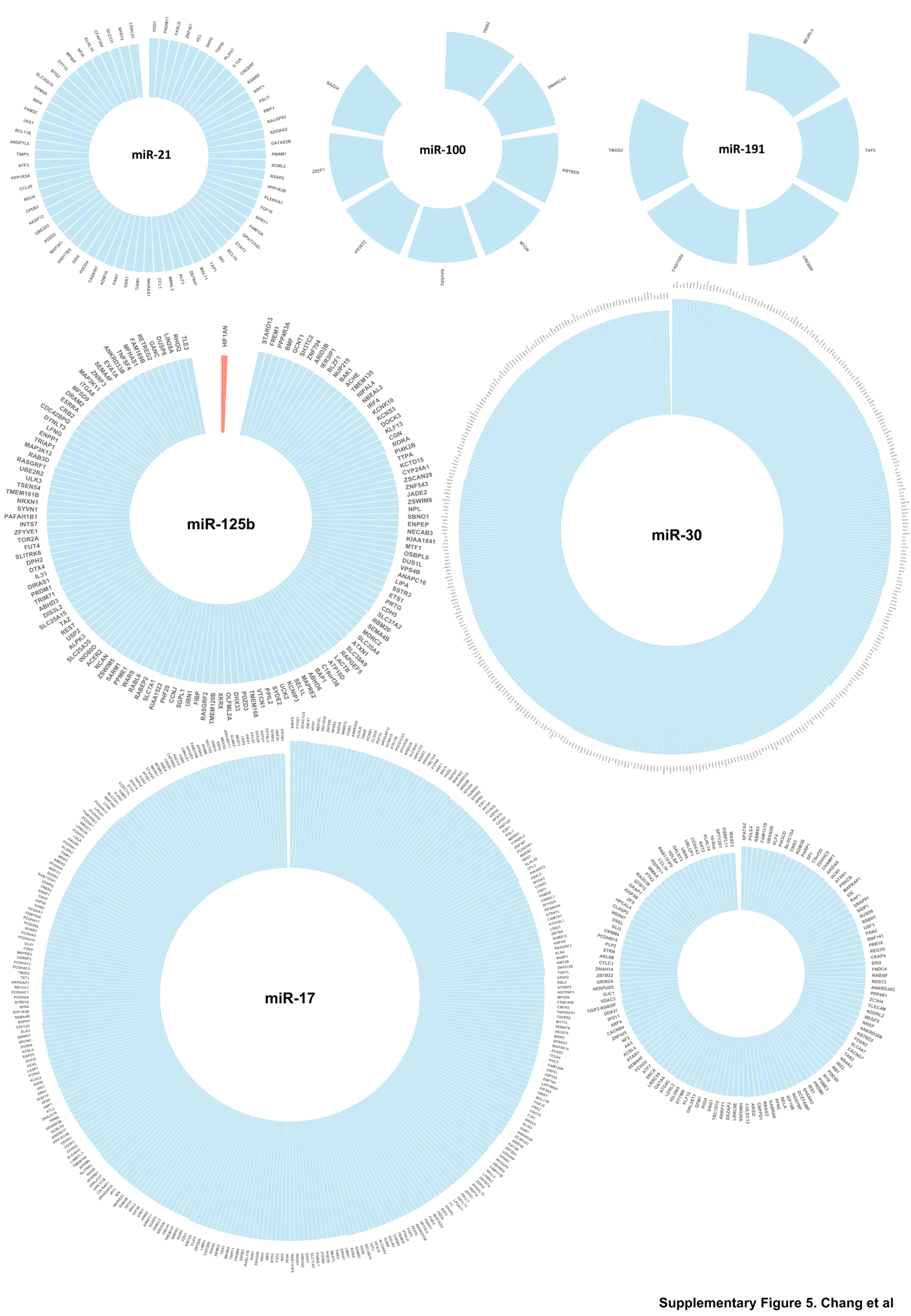


**Supplementary Figure 5.** A zoom-in image of direct target genes of miRNAs of interest, shown in Figure 5D

**
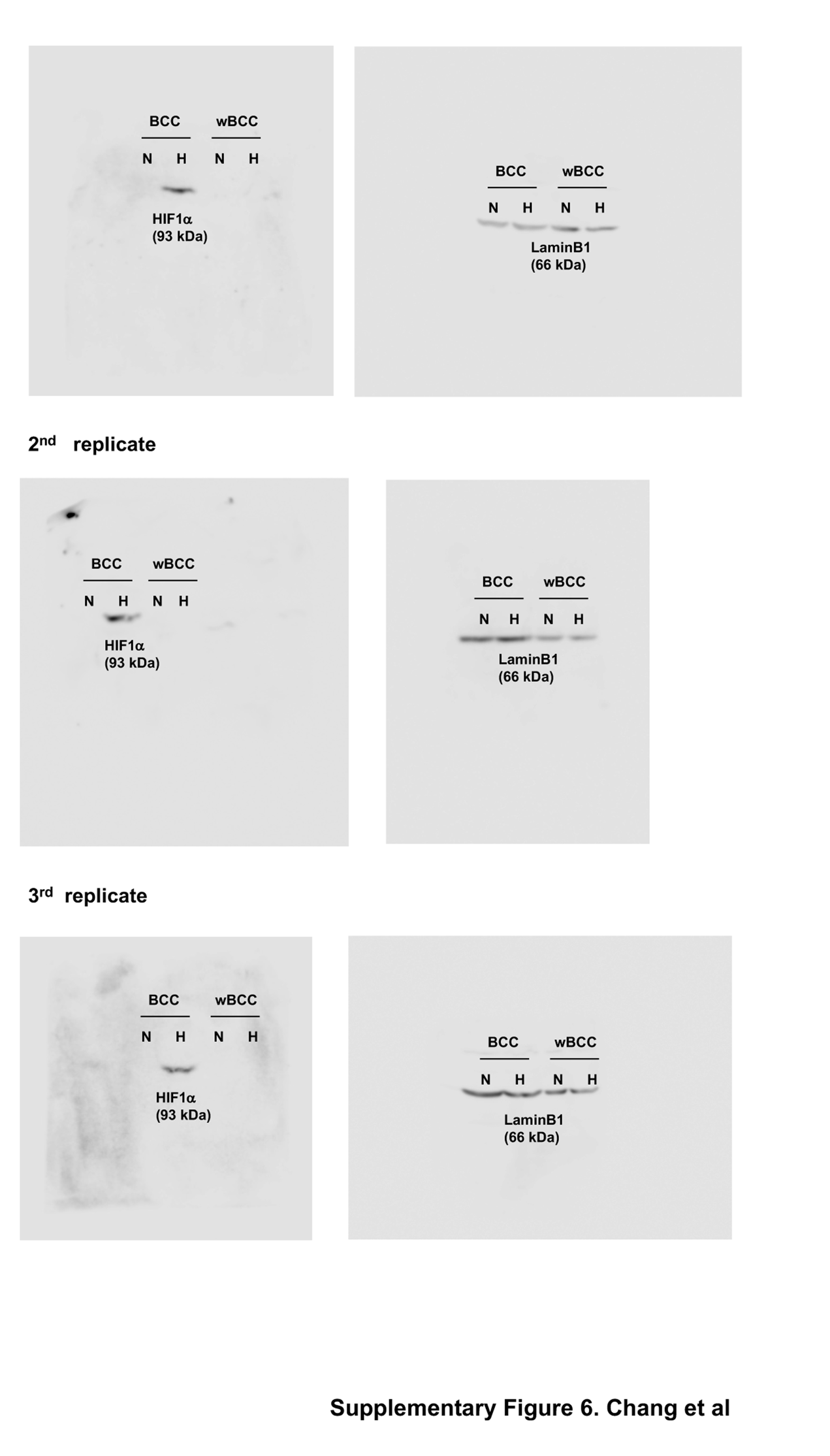
Supplementary Figure 6. Full-length blots of Western blot images.** HIF1α expression in BCC and wBCC shown in Figure 5F.

**Supplementary Table 1. A systematic review of miRNAs of interest which directly inhibit HIF1α expression**

| **Year/Author** | **miRNA** | **Effects on HIF1α** | **Cell type** |
| --- | --- | --- | --- |
| 2019/Chi Yang ^1^ | miR-21 | Induce | Human umbilical cord blood-derived mesenchymal stem cells |
| 2021/Jing Sun ^2^ | miR-21 | Induce | Human fetal hepatocytes |
| 2021/[Suvi Linna-Kuosmanen](https://pubmed.ncbi.nlm.nih.gov/?term=Linna-Kuosmanen+S&cauthor_id=32683448) ^3^ | miR-21  miR-100 | Induce  Inhibit | Human umbilical vein endothelial cells (HUVEC) |
| 2019/[Yongchun Zhang](https://pubmed.ncbi.nlm.nih.gov/?term=Zhang+Y&cauthor_id=31128066) ^4^ | miR-21 | Induce | HUVEC |
| 2011/[Ling-Zhi Liu](https://pubmed.ncbi.nlm.nih.gov/?term=Liu+LZ&cauthor_id=21544242) ^5^ | miR-21 | Induce | Human prostate cancer cells |
| 2018/Nana Song ^6^ | miR-21 | Induce | Human renal proximal tubular cells |
| 2019/Xueqin Zhang ^7^ | miR-21 | Induce | Human periodontal ligament cells |
| 2019/ Yun Zhang ^8^ | miR-21 | Induce | Mouse corneal epithelial cells |
| 2020/ Jin-Rui Liu ^9^ | miR-21 | Induce | Human proximal tubular cells |
| 2016/ Lili Song ^10^ | miR-21 | Induce | Human cervical cancer cells |
| 2014/ [Adèle Richart](https://pubmed.ncbi.nlm.nih.gov/?term=Richart+A&cauthor_id=25069679) ^11^ | miR-21 | Induce | Human embryonic stem cells |
| 2012/ Mingli Han ^12^ | miR-21 | Induce | Human breast cancer stem cells |
| 2019/ Tsai-Kun Wu ^13^ | miR-21 | Induce | Human clear cell renal carcinoma cells |
| 2022/ Xiaoran Xie ^14^ | miR-21 | Induce | HUVEC |
| 2017/ Xioyan Jiao ^15^ | miR-21 | Induce | Human proximal tubular cells |
| 2016/ Shumei Jiang ^16^ | miR-21 | Induce | Human non‑small cell lung cancer cells |
| 2019/ Yang An ^17^ | miR-21 | Induce | Rat adipose tissue-derived mesenchymal stem cells |
| 2013/ Ping Jia ^18^ | miR-21 | Induce | Mouse kidneys |
| 2017/ [Rashidul Haque](https://pubmed.ncbi.nlm.nih.gov/?term=Haque+R&cauthor_id=28465657) ^19^ | miR-21 | Induce | Human retinal pigment epithelium cell |
| 2016/ Yong Zhou ^20^ | miR-21 | Induce | Human umbilical cord blood -derived mesenchymal stem cells |
| 2012/ Bin Bao ^21^ | miR-21 | Induce | Human pancreatic cancer cells |
| 2022/ Yanyi Sun ^22^ | miR-21 | Induce | Mouse B cells |
| 2017/ Yun Zhang ^23^ | miR-21 | Induce | Mouse cornea |
| 2015/ Yang ^24^ | miR-21 | Induce | Human Bladder cancer cells |
| 2022/ Zheng ^25^ | miR-21 | Induce | Human Glioma cells |
| 2019/ Sun ^26^ | miR-21 | Induce | Human pancreatic cancer cells |
| 2018/ Sheng ^27^ | miR-21 | Induce | Rat vertebral pulp and annulus fibrosus |
| 2017/ Zhang ^28^ | miR-21 | Induce | Human adipose tissue-derived mesenchymal stem cells |
| 2013/ Zhao ^29^ | miR-21 | Induce | Murine breast cancer cells |
| 2013/ Xie ^30^ | miR-21 | Induce | Human ovarian cancer cells |
| 2019/ Kilari ^31^ | miR-21 | Induce | Mouse arteriovenous fistula |
| 2021/Shunichi Oka ^32^ | miR-21 | Inhibit | Mouse pre-osteoblast cells |
| 2014/ Yang Liu ^33^ | miR-21 | Induce | Human cardiomyocytes |
| 2016/ Cheng ^34^ | miR-17 | Induce | Human pulmonary artery smooth muscle cells |
| 2021/ Xiyang Wei ^35^ | miR-125b | Inhibit | Human hepatocellular carcinoma cells |
| 2021/ PengLi Wang ^36^ | miR-125b | Inhibit | Human pancreatic adenocarcinoma cells |
| 2013/ Jun He ^37^ | miR-125b | Inhibit | Human ovarian cancer cells |
| 2018/ Paola Maroni ^38^ | miR-125b | Inhibit | Human breast cancer cells |
| 2022/ Jin-xia Liu ^39^ | miR-125b | Inhibit | Human retinal pigment epithelium cells |
| 2021/ Wang ^40^ | miR-125b | Inhibit | Human pancreatic cancer cells |
| 2013/ C Blick ^41^ | miR-100 | Inhibit | Human bladder cancer cells |

**References used for systematic review**

1. Yang, C. *et al.* MiRNA-21 promotes osteogenesis via the PTEN/PI3K/Akt/HIF-1α pathway and enhances bone regeneration in critical size defects. *Stem Cell Research and Therapy* **10**, (2019).

2. Sun, J. *et al.* microRNA-21, via the HIF-1α/VEGF signaling pathway, is involved in arsenite-induced hepatic fibrosis through aberrant cross-talk of hepatocytes and hepatic stellate cells. *Chemosphere* **266**, (2021).

3. Linna-Kuosmanen, S. *et al.* NRF2 is a key regulator of endothelial microRNA expression under proatherogenic stimuli. *Cardiovascular Research* **117**, 1339–1357 (2021).

4. Zhang, Y. *et al.* Ionizing radiation-inducible microRNA-21 induces angiogenesis by directly targeting PTEN. *Asian Pacific Journal of Cancer Prevention* **20**, 1587–1593 (2019).

5. Liu, L. Z. *et al.* Mir-21 induced angiogenesis through AKT and ERK activation and HIF-1α expression. *PLoS ONE* **6**, (2011).

6. Song, N. *et al.* miR-21 protects against ischemia/reperfusion-induced acute kidney injury by preventing epithelial cell apoptosis and inhibiting dendritic cell maturation. *Frontiers in Physiology* **9**, (2018).

7. Zhang, X. *et al.* Effect of microRNA‑21 on hypoxia‑inducible factor‑1α in orthodontic tooth movement and human periodontal ligament cells under hypoxia. *Experimental and Therapeutic Medicine* (2019) doi:10.3892/etm.2019.7248.

8. Zhang, Y. *et al.* The role of the MIR-21/SPRY2 axis in modulating proangiogenic factors, epithelial phenotypes, and wound healing in corneal epithelial cells. *Investigative Ophthalmology and Visual Science* **60**, 3854–3862 (2019).

9. Jin-rui Liu *et al.* Caloric restriction alleviates aging-related fibrosis of kidney through downregulation of miR-21 in extracellular vesicles. *Aging (Albany NY)* **12**, 18052–18072 (2020).

10. Song, L. *et al.* MiR-21 modulates radiosensitivity of cervical cancer through inhibiting autophagy via the PTEN/Akt/HIF-1α feedback loop and the Akt-mTOR signaling pathway. *Tumor Biology* **37**, 12161–12168 (2016).

11. Richart, A. *et al.* MicroRNA-21 coordinates human multipotent cardiovascular progenitors therapeutic potential. *Stem Cells* **32**, 2908–2922 (2014).

12. Han, M. *et al.* MiR-21 regulates epithelial-mesenchymal transition phenotype and hypoxia-inducible factor-1α expression in third-sphere forming breast cancer stem cell-like cells. *Cancer Science* **103**, 1058–1064 (2012).

13. Wu, T. K. *et al.* The uremic toxin p-cresyl sulfate induces proliferation and migration of clear cell renal cell carcinoma via microRNA-21/ HIF-1α axis signals. *Scientific Reports* **9**, (2019).

14. Xie, X. *et al.* Circulating exosomal miR-21 mediates HUVEC proliferation and migration through PTEN/PI3K/AKT in Crohn’s disease. *Annals of Translational Medicine* **10**, 258–258 (2022).

15. Jiao, X. *et al.* miR-21 contributes to renal protection by targeting prolyl hydroxylase domain protein 2 in delayed ischaemic preconditioning. *Nephrology* **22**, 366–373 (2017).

16. Jiang, S. *et al.* MicroRNA-21 modulates radiation resistance through upregulation of hypoxia-inducible factor-1α-promoted glycolysis in non-small cell lung cancer cells. *Molecular Medicine Reports* **13**, 4101–4107 (2016).

17. An, Y. *et al.* Exosomes from Adipose-Derived Stem Cells (ADSCs) Overexpressing miR-21 Promote Vascularization of Endothelial Cells. *Scientific Reports* **9**, (2019).

18. Jia, P. *et al.* MiR-21 contributes to xenon-conferred amelioration of renal ischemia-reperfusion injury in mice. *Anesthesiology* **119**, 621–630 (2013).

19. Rashidul Haque *et al.* The MicroRNA-21 signaling pathway is involved in prorenin receptor (PRR) –induced VEGF expression in ARPE-19 cells under a hyperglycemic condition . *Molecular Vision* **23**, 251–262 (2017).

20. Zhou, Y. *et al.* Human Stem Cells Overexpressing MIR-21 Promote Angiogenesis in Critical Limb Ischemia by Targeting CHIP to Enhance HIF-1α Activity. *Stem Cells* **34**, 924–934 (2016).

21. Bao, B. *et al.* Hypoxia-Induced Aggressiveness of Pancreatic Cancer Cells Is Due to Increased Expression of VEGF, IL-6 and miR-21, Which Can Be Attenuated by CDF Treatment. *PLoS ONE* **7**, (2012).

22. Sun, Y. *et al.* Splenic Marginal Zone B Lymphocytes Regulate Cardiac Remodeling After Acute Myocardial Infarction in Mice. *J Am Coll Cardiol* **79**, 632–647 (2022).

23. Zhang, Y., Zhang, T., Ma, X. & Zou, J. *Subconjunctival injection of antagomir-21 alleviates corneal neovascularization in a mouse model of alkali-burned cornea*. www.impactjournals.com/oncotarget (2016).

24. Yang, X. *et al.* A lentiviral sponge for miRNA-21 diminishes aerobic glycolysis in bladder cancer T24 cells via the PTEN/PI3K/AKT/mTOR axis. *Tumor Biology* **36**, 383–391 (2015).

25. Zheng, T. *et al.* Anti-MicroRNA-21 Oligonucleotide Loaded Spermine-Modified Acetalated Dextran Nanoparticles for B1 Receptor-Targeted Gene Therapy and Antiangiogenesis Therapy. *Advanced Science* **9**, (2022).

26. Sun, J. *et al.* Downregulation of miR-21 inhibits the malignant phenotype of pancreatic cancer cells by targeting VHL. *OncoTargets and Therapy* **12**, 7215–7226 (2019).

27. Sheng, X., Guo, Q., Yu, J. & Xu, Y. Experimental research on the effect of microRNA-21 inhibitor on a rat model of intervertebral disc degeneration. *Experimental and Therapeutic Medicine* **16**, 67–72 (2018).

28. Zhang, X. *et al.* In situ forming hydrogels with long-lasting miR-21 enhances the therapeutic potential of MSC by sustaining stimulation of target gene. *Journal of Biomaterials Science, Polymer Edition* **28**, 1639–1650 (2017).

29. Zhao, D. *et al.* In Vivo Monitoring of Angiogenesis Inhibition via Down-Regulation of Mir-21 in a VEGFR2-Luc Murine Breast Cancer Model Using Bioluminescent Imaging. *PLoS ONE* **8**, (2013).

30. Xie, Z., Cao, L. & Zhang, J. miR-21 modulates paclitaxel sensitivity and hypoxia-inducible factor-1α expression in human ovarian cancer cells. *Oncology Letters* **6**, 795–800 (2013).

31. Kilari, S. *et al.* The Role of MicroRNA-21 in Venous Neointimal Hyperplasia: Implications for Targeting miR-21 for VNH Treatment. *Molecular Therapy* **27**, 1681–1693 (2019).

32. Oka, S. *et al.* MicroRNA-21 facilitates osteoblast activity. *Biochemistry and Biophysics Reports* **25**, (2021).

33. Liu, Y. *et al.* A feedback regulatory loop between HIF-1α and miR-21 in response to hypoxia in cardiomyocytes. *FEBS Letters* **588**, 3137–3146 (2014).

34. Chen, T. *et al.* MiR-17/20 controls prolyl hydroxylase 2 (PHD2)/hypoxia-inducible factor 1 (HIF1) to regulate pulmonary artery smooth muscle cell proliferation. *J Am Heart Assoc* **5**, (2016).

35. Wei, X. *et al.* MiR-125b Loss Activated HIF1α/ pAKT Loop, Leading to Transarterial Chemoembolization Resistance in Hepatocellular Carcinoma. *Hepatology* **73**, 2021 (2020).

36. Wang, P., Zheng, D., Qi, H. & Gao, Q. Thioredoxin-interacting protein is a favored target of miR-125b, promoting metastasis and progression of pancreatic cancer via the HIF1α pathway. *Journal of Biochemical and Molecular Toxicology* **35**, (2021).

37. He, J. *et al.* Roles and Mechanism of miR-199a and miR-125b in Tumor Angiogenesis. *PLoS ONE* **8**, (2013).

38. Maroni, P., Bendinelli, P., Matteucci, E. & Desiderio, M. A. The therapeutic effect of MIR-125b is enhanced by the prostaglandin endoperoxide synthase 2/cyclooxygenase 2 blockade and hampers ETS1 in the context of the microenvironment of bone metastasis. *Cell Death and Disease* **9**, (2018).

39. Liu, J. xia *et al.* MiR-125b attenuates retinal pigment epithelium oxidative damage via targeting Nrf2/HIF-1α signal pathway. *Experimental Cell Research* **410**, (2022).

40. Wang, P., Zheng, D., Qi, H. & Gao, Q. MiR-125b enhances metastasis and progression of cancer via the TXNIP and HIF1 pathway in pancreatic cancer. *Cancer Biomarkers* **31**, 27–38 (2021).

41. Blick, C. *et al.* Hypoxia regulates FGFR3 expression via HIF-1α and miR-100 and contributes to cell survival in non-muscle invasive bladder cancer. *British Journal of Cancer* **109**, 50–59 (2013).
